# Urinary DNA-methylation and protein biomarkers identify urothelial carcinoma among other genitourinary diseases and cancer-free individuals

**DOI:** 10.1186/s12967-024-05844-x

**Published:** 2024-11-26

**Authors:** Kerstin Lang, Christina U. Köhler, Katharina Wichert, Thomas Deix, Georg Bartsch, Gudrun Sommer, Christiane Lübke, Florian Roghmann, Moritz J. Reike, Harald Krentel, Katja Engellandt, Sven Schiermeier, Valentin Menke, Joachim Noldus, Thomas Behrens, Thomas Brüning, Heiko U. Käfferlein

**Affiliations:** 1 Institute for Prevention and Occupational Medicine of the German Social Accident Insurance, Institute of the Ruhr University Bochum (IPA), 44789 Bochum, Germany; 2 URO DR, Clinic of Urology and Andrology, Mülheimer Str. 37, 40878 Ratingen, Germany; 3 Surgery of Gynecology, Husemannplatz 6-7, 44787 Bochum, Germany; 4 Department of Urology, Marien Hospital Herne, Ruhr University Bochum, Hölkeskampring 40, 44625 Herne, Germany; 5 Department of Obstetrics and Gynecology, Bethesda Hospital, Duisburg, Germany; 6 Department of Obstetrics and Gynecology, Marien-Hospital, University Witten-Herdecke, Witten, Germany; 7 Department of Obstetrics and Gynecology, St. Anna Hospital, Herne, Germany

**Keywords:** Bladder cancer, Verification, Urine, Biomarkers, DNA methylation

## Abstract

**Background:**

For more than 80 years, cystoscopy has been the gold standard for identification of urothelial carcinoma (UCa). Because of many factors, such as pain of the patients during this procedure or the costs involved, non-invasive detection of UCa remains a challenge. Herein, we verify our previously identified urinary biomarkers C-X-C Motif Chemokine Ligand 16 (CXCL16) and transforming growth-factor beta induced protein (TGFBI) on the protein level as well as the CpG sites ALOX5, TRPS1 and an intergenic region on Chromosome 16 on DNA methylation level in an independent cross-sectional study.

**Methods:**

We collected N = 1119 urines from individuals coming to urological and gynecological check-ups, follow-up care or patients suspicious for UCa or already diagnosed for different urologic or gynecologic cancer entities. We performed methylation analysis of various CpG sites with DNA isolated from urine sediment and quantified the concentration of the protein markers CXCL16 and TGFBI in the corresponding urine supernatant using ELISA. We tested for patient-group differences with two-sided Wilcoxon rank sum tests and examined the performance with receiver operating characteristic curves. For verification, we analyzed the marker performance with previously set cutoff-values and marker combinations with established and experimental algorithms (with logical OR-conjunction, iterative threshold-based biomarker and score combining algorithm “PanelomiX”).

**Results:**

Evaluation confirmed that our previously identified protein and DNA methylation biomarkers can distinguish UCa from frequent urological and gynecological cancers. CXCL16 and TGFBI discriminated UCa cases with a sensitivity of 31% and 56% and a specificity of 94% and 85%, respectively. Combining methylation markers resulted in UCa detection in men with a sensitivity of 54% and a specificity of 94%. Extending analysis by combining all methylation and protein markers (up to five markers in total) yielded a convincingly high specificity of 97% at a sensitivity of 72% for the identification of UCa patients within a heterogeneous collective of cancer-free individuals and patients suffering from urological or gynecological cancers.

**Conclusion:**

Combining various biomarkers at protein and DNA level demonstrates a new option of non-invasive UCa diagnosis in urine, and thus might help to reduce the number of unnecessary cystoscopies, especially in patients without a history of UCa.

**Supplementary Information:**

The online version contains supplementary material available at 10.1186/s12967-024-05844-x.

## Background

With approximately 30,000 new cases per year, urothelial cancer (UCa) is one of the most frequent tumors in Germany [1]. Men are affected about three times more than women. UCa is a prognostically favorable cancer if diagnosed at an early stage with a 5-year survival rate of approximately 75%. However, UCa shows a high tendency for recurrence. From an economic point of view, UCa is the most expensive form of all cancers, considering the time from the initial diagnosis to patient death, due to short-intervalled follow-up examinations [2]. A common symptom of bladder cancer is usually painless, micro- or macrohaematuria that can easily be identified by urine stick tests or even visual inspection of the urine. However, haematuria can also occur in benign diseases of the urinary tract such as kidney or bladder infections or nephrolithiasis and thus is not specific for urothelial cancer [3].

Urethrocystoscopy, even though invasive, is the gold standard for diagnosing UCa. However, cystoscopy can be painful and might provoke discontinuation of follow-up visits. In turn, this might lead to delayed detection of recurrencies and cancer progression, which could have negative impact on patients’ prognosis [5, 6]. In addition, cystoscopy often misses flat and aggressive tumors (about 20–30% of UCa) such as carcinomas in situ (CIS), which are difficult to distinguish from inflammation of the bladder mucosa [7].

So far, non-invasive methods such as cytology are only considered “add-on” investigations [8]. In example, cytology has a high specificity (approximately 90%), but a low sensitivity (approximately 30%; [4]). Other non-invasive methods such as UCa-associated biomarkers are not part of the decision-making process at all. However, clinically established biomarkers with a sufficiently high specificity and acceptable sensitivity (> 90% specificity, > 50% sensitivity), if regularly measured, could reduce painful cystoscopies to those cases in which the marker was conspicuous.

The few diagnostic biomarkers used to date for the detection of UCa focus on tumor-associated antigens or genetic modifications (amplifications, deletions) in exfoliated urothelial cells [9]. Some of them have already been approved by the U.S. Food and Drug Administration (FDA) (e.g. NMP22® Bladder Chek®, BTATRAK™) [10, 11], yet none of these tumor markers have been included in guidelines of urologic societies. This may mainly be due to the fact that most testing methods are associated with a high rate of false-positive findings and thus reduced specificity, especially in the presence of haematuria, urinary tract infection or nephrolithiasis [12, 13]. For UCa detection, NMP22 showed a sensitivity of 52–59% in various studies and a specificity of 87–89% [14]. Bladder tumor antigen (BTA) tests are approved for bladder cancer surveillance in addition to cystoscopy with varying sensitivities from 54 to 61% and specificities ranging from 74 to 86%, respectively [15, 16].

In addition to the aforementioned proteins (e.g. NMP22, BTA), former studies demonstrated that DNA methylation of bladder epithelium in UCa patients showed marked abnormalities compared to non-UCa control subjects [17, 18]. A great variability in sensitivity and specificity across studies has been previously observed, and even the most-promising methylation biomarkers according to a most recent meta-analysis displayed variations between 10 and 15% as far as the sensitivity and of up to 50% as far as specificity was concerned [19]. This variability, among other reasons, is mainly due to methodological differences and heterogeneities in the studied collectives such as age, gender, and different ratios between individuals with and without cancer [12, 19]. Urinary DNA methylation targets have been widely discussed as biomarkers for UCa [20–22], too. Very recently, the first urine biomarker methylation test Bladder EpiCheck® received FDA clearance for monitoring of non-muscle invasive bladder cancer (NMIBC) recurrence in conjunction with cytology [23]. Several studies analyzing the performance of Bladder EpiCheck® test showed a sensitivity of approximately 90% and a specificity of 88% in patients with high grade NMIBC under surveillance [23, 24]; however, the sensitivity is markedly reduced to approximately 67% in patient collectives including low-grade-patients We also identified numerous urinary biomarkers on both the protein and DNA methylation level. These include TGFBI and CXCL16 on the protein level [25, 26] and ALOX5, TRPS1 and an intergenic region on Chromosome 16 on the methylation level [27, 28]. Here, we report the verification of these markers in an independent cross-sectional study collective called “UroSpec”. For this purpose, we collected urine from individuals that presented themselves for regular urological and gynecological preventive medical check-ups or for urine-related symptoms (e.g. frequent or painful urination, microhaematuria); we also included patients suspicious for or already diagnosed with urologic or gynecologic cancers. We studied the usefulness of a marker panel combining protein and methylation markers for further improving UCa diagnosis.

## Materials and methods

### Study population and collection of samples

To verify our original findings that a newly identified set of methylation biomarkers as well as the two proteins TGFBI and CXCL16 can be used for diagnosing UCa in urine [25–28], we established a cross-sectional study. Therefore, we collected from October 2014 to March 2020 urine in various physicians’ offices (urologists and gynecologists), and in four hospitals with a Department for Urology or a Department for Gynecology. All institutions were pre-selected because of their location (< 50 km) to our institute, and they volunteered to participate in our study. We collected urine on a daily basis from all patients presenting to urological and gynecological preventive medical check-ups, individuals with all types of urinary symptoms (e.g. frequent or painful urination; microhaematuria), or patients suspicious for genitourinary cancers as well as patients already diagnosed for an urologic (prostate, urothelium, kidney) or gynecologic cancer (breast, ovarian, cervix, uterus, vagina) coming in for follow-up care. The final diagnosis in all patients was pathologically confirmed as part of the usual clinical routine in the respective hospitals. We excluded persons younger than 40 years of age and patients undergoing current chemo- or radiotherapy. From every individual participating in our study, we collected a letter of informed consent, conducted a questionnaire (to document e.g., gender, age, smoking status, other diseases, medication etc.), documented the actual findings of the physician and/or the pathologist, and generated a case report to the urine collection (including e.g., timepoint of collection, urine parameters such as number of leukocytes, creatinine etc.). Characteristics of our study sample are summarized in Table 1. The study was approved by the ethics review board of the Ruhr-University Bochum, Germany (No. 4785-13) and all participants gave written informed consent. In our previous studies, we found that DNA methylation markers showed a better performance in men than in women and that leukocyte counts > 500/µL impede urinary UCa-detection [27]. Therefore, we analyzed different subpopulations and focussed our analysis of methylation markers on the male individuals among the study participants with urinary leukocyte counts < 500/µL (i.e. 522 individuals, Table 1). We also stratified patients for their UCa history when calculating the diagnostic performance of our markers or marker combinations because in previous collectives we found tumors of patients under UCa surveillance to be less potently detected [27, 28].

**Table 1:** Description of the verification collective investigated for DNA methylation and protein biomarkers, respectively

	Methylation biomarkers (N = 522)			Protein biomarkers (N = 1119)		
	UCa	Other cancer	No cancer	UCa	Other cancer	No cancer
	N (%)	N (%)	N (%)	N (%)	N (%)	N (%)
Total	54	96	372	71	207	841
Age (years)						
< 70	26 (48.1)	53 (55.2)	189 (50.8)	33 (46.5)	125 (60.4)	508 (60.4)
≥ 70	28 (51.9)	43 (44.8)	181 (48.7)	38 (53.5)	79 (38.2)	328 (39.0)
Unknown	0 (0.0)	0 (0.0)	2 (0.5)	0 (0.0)	3 (1.0)	5 (0.6)
Sex						
Male	54 (100.0)	96 (100.0)	372 (100.0)	62 (87.3)	118 (57.0)	473 (56.2)
Female	–	–	–	9 (12.7)	89 (43.0)	368 (43.8)
Leucocytes						
Negative	32 (59.3)	79 (82.3)	312 (83.9)	39 (54.9)	142 (68.6)	594 (70.6)
~ 10	0 (0.0)	0 (0.0)	0 (0.0)	0 (0.0)	1 (0.5)	0 (0.0)
~ 25	9 (16.7)	11 (11.5)	37 (9.9)	12 (16.9)	21 (10.1)	88 (10.5)
~ 100	13 (24.1)	6 (0.63)	23 (6.2)	15 (21.1)	21 (10.1)	71 (8.4)
~ 500	–	–	–	5 (7.0)	20 (9.7)	87 (10.3)
Unknown	0 (0.0)	0 (0.0)	0 (0.0)	0 (0.0)	2 (1.0)	1 (0.1)
Erythrocytes						
Negative	22 (40.7)	73 (76.0)	260 (69.9)	28 (39.4)	147 (71.0)	511 (60.8)
~ 10	5 (9.3)	12 (12.5)	38 (10.2)	5 (7.0)	22 (10.6)	103 (12.2)
~ 25	3 (5.6)	4 (4.2)	33 (8.9)	4 (5.6)	10 (4.8)	80 (9.5)
~ 50	9 (16.7)	1 (1.0)	18 (4.8)	10 (14.1)	12 (5.8)	56 (6.7)
~ 250	15 (27.8)	6 (0.63)	23 (6.2)	24 (33.8)	14 (6.8)	89 (10.6)
Unknown	0 (0.0)	0 (0.0)	0 (0.0)	0 (0.0)	2 (1.0)	2 (0.2)
UCa-history						
No	26 (48.1)	93 (96.9)	342 (91.9)	33 (46.5)	200 (96.6)	786 (93.5)
Yes	28 (51.9)	3 (3.1)	30 (8.1)	38 (53.5)	5 (2.4)	52 (6.2)
Unknown	0 (0.0)	(0.0)	0 (0.0)	0 (0.0)	2 (1.0)	3 (0.4)
UCa tumor grading						
Low grade	23 (42.6)	–	–	28 (39.4)	–	–
High-grade	13 (24.1)	–	–	16 (22.5)	–	–
Unknown	18 (33.3)	–	–	27 (38.0)	–	–
Other current cancers*						
Breast cancer	0 (0.0)	0 (0.0)	–	0 (0.0)	40 (19.3)	–
Ovarian cancer	–	–	–	0 (0.0)	8 (3.9)	–
Cervical cancer	–	–	–	0 (0.0)	11 (5.3)	–
Prostate cancer	1 (1.9)	76 (79.2)	–	1 (1.4)	94 (45.4)	–
Kidney cancer	3 (5.6)	22 (22.9)	–	6 (8.5)	40 (19.3)	0 (0.0)
Other urol. cancer**	8 (14.8)	0 (0.0)	–	11 (15.5)	0 (0.0)	–
Other gyn. cancer**	–	–	–	0 (0.0)	17 (8.2)	–

Urothelial carcinoma (UCa), documented cancers except UCa (other cancer) and individuals without current malignant diseases (no cancer) are listed. Case counts are given regarding age and gender, UCa-history, UCa tumor grading and urinary blood cell counts. In addition, the entity of non UCa cancers is given. *Can include multiple cancers **excluding the above mentioned cancers

### Urine sample preparation

Urine samples were collected in the surgery or hospital during the morning, and then transported to our laboratory. Upon arrival, urinary leukocytes (~ 0, ~ 25, ~ 100, ~ 500 leukocytes/µL urine) and number of erythrocytes (negative: ~ 10, ~ 25–50, ~ 150–250 erythrocytes/µL urine) were measured using Combur-Test® sticks (Roche, Mannheim, Germany). Creatinine concentration was determined according to Jaffé [29]. Urine was centrifuged (10 min at 1700×*g* at 10 °C) and the supernatant was stored at − 80 °C. The sediment was then washed with phosphate buffered saline (PBS), centrifuged again (4000×*g*, 10 min) and the cell pellet was stored at − 80 °C.

### DNA isolation from urine

The preparation of DNA has been described in our previous study in detail [27]. In brief, DNA was isolated from the cell pellet by using the QIAmp MinElute Virus Spin Kit (Qiagen, Hilden, Germany). After the digestion of RNA with DNAse-free RNAse (Roche, Mannheim, Germany), DNA was purified by the Clean and Concentrator TM-25 Kit (Zymo Research, Corporation, Irvine, CA, USA), eluted using TE buffer (AppliChem, Darmstadt, Germany), and stored at − 20 °C until further analysis.

### Quantitative mass spectrometry of DNA methylation

A minimum of 200 ng of urinary DNA was bisulfite-converted by the EZ DNA Methylation Gold Kit (Zymo Research, Orange, CA, USA) according to the recommendations of the manufacturer. DNA methylation was assessed by matrix-assisted laser desorption/ionization–time-of-flight (MALDI-TOF) mass spectrometry (MassARRAY EpiTYPER system, Agena Bioscience GmbH, Hamburg, Germany) enabling the quantitative measurement of CpG methylation at single dinucleotide resolution [30]. All analyses were carried out according to the protocol of Agena Bioscience GmbH and have been previously described in detail [27]. In brief, bisulfite-converted DNA underwent PCR at a uniform annealing temperature of 56 °C and using primers that had previously been designed by Agena’s EpiDesigner software (http://www.epidesigner.com/index.html) to specifically amplify the amplicons of interest [27]. In the cited manuscript, ALOX5 is referred to as Amplicon02_CpG6 (with the C of interest in position Chr10:45,418,979 according to Genome build GRCh38/hg38), TRPS1 is referred to Amplicon 35_CpG7 (with the C of interest in position Chr8:115,667,708 according to Genome build GRCh38/hg38), and Chromosome 16 is referred to as Amplicon 78_CpG_2.3 (with the Cs of interest in positions Chr16: 51,153,010 and Chr16: 51,153,014 according to Genome build GRCh38/hg38). After shrimp alkaline phosphatase treatment, PCR products were subjected to in vitro transcription. Finally, RNA cleavage products were measured by mass spectrometry.

### Enzyme-linked immunosorbent assay

For quantification of βIGH3/TGFBI in urine supernatant we used the human βIGH3 DuoSet ELISA Kit (Biotechne, MN, USA), and for quantification of CXCL16 the custom human CXCL16 ELISA Kit (RayBiotech, GA, USA) as described previously [25, 26]. All samples were measured in duplicate and confirmed in two independent experiments. Standardization by urinary creatinine concentration was obtained by dividing the TGFBI or CXCL16 concentration (pg/mL) of a particular urine sample by its corresponding creatinine level (mg/mL), such that normalized TGFBI or CXCL16 levels are reported in units of pg/mg creatinine.

### Statistical analysis

We created boxplots for each marker’s concentration distribution by patient group. Marker differences between patient groups were tested with two-sided Wilcoxon rank sum tests at a level of significance of 5%. We used receiver operating characteristic (ROC) curves to examine each marker’s sensitivity and specificity for the classification of UCa. In order to verify the biomarkers, their performances with the known thresholds from the identification studies were evaluated. We used sensitivity and specificity as performance measures as they are unbiased by class imbalance [31].

Thinking one step further, we decided to combine both analysis methods at DNA and protein level to evaluate if the diagnostic output of urine analysis can be improved. For this purpose, we analyzed the overall patient collective using different approaches. Accordingly, for analysis of biomarker combinations, we used Boolean (logic) “OR” conjunctions. With the OR conjunction, a patient is classified as positive, if at least one biomarker exceeded its threshold. If the patient has missing values in at least one marker and the remaining markers were all negative, the patient was not classified. Again, we used the known thresholds from the identification studies for the OR-combinations. We used Venn-Diagrams to visualize the overlap of positive markers. Additionally, we conducted the experimental iterative threshold-based biomarker and score combining algorithm “PanelomiX” [32]. PanelomiX identifies the best performing biomarker panels for the classification of UCa according to a selected criterion (constraint on sensitivity, specificity, or accuracy). We determined a specificity of at least 95%. The optimization algorithm is described elsewhere [32]. In brief, at first, candidate thresholds for each marker in the panel were selected, based on the observed local extrema of the ROC curve. Next, an exhaustive search algorithm was applied to optimize the panel. For all combinations of biomarkers and their individual threshold values, the patients’ score was calculated as the number of biomarkers that exceeded their thresholds. A patient was then classified as positive, if his/her score equals or exceeds the score threshold, and subsequently the panel’s sensitivity and specificity was calculated. Therefore, the PanelomiX algorithm calculates new data-based thresholds for the panel. For the panel verification, we used a stratified 10 × threefold cross-validation [33]. For this purpose, our data was randomly split into three data subsets considering the distribution of the UCa status in the original data set. Successively, each of the three subsets (two training sets, one test set) is excluded as test set. The best panel is then selected for the remaining two training sets and its performance is calculated on the test set. This procedure is repeated ten times. The final performance is then calculated as mean performance across all ten test sets.

We used the statistical software R, version 4.2.2 for all calculations (R Core Team; [34]). For the combination with PanelomiX, we used the experimental R-package “PanelomiX” via GitHub (https://github.com/xrobin/PanelomiX).

Figures were created using GraphPad Prism, version 9.

## Results

### Study population characteristics

The main characteristics of the study group are depicted in Table 1. In summary, we evaluated 71 UCa cases, 207 other cancer cancers and 841 control subjects without cancer. 58% of the subjects were men and 42% women, and 60% of the participants were 70 years of age or older. From all UCa cases—independent from UCa history—39% cases were low grade, 23% high grade and for 38% grading was unknown. In the group of other cancers 36% were gynecological and 64% other urological cancers.

### Thresholds to identify UCa obtained from the identification approach can also be applied to the “UroSpec” collective

**Fig. 1:**
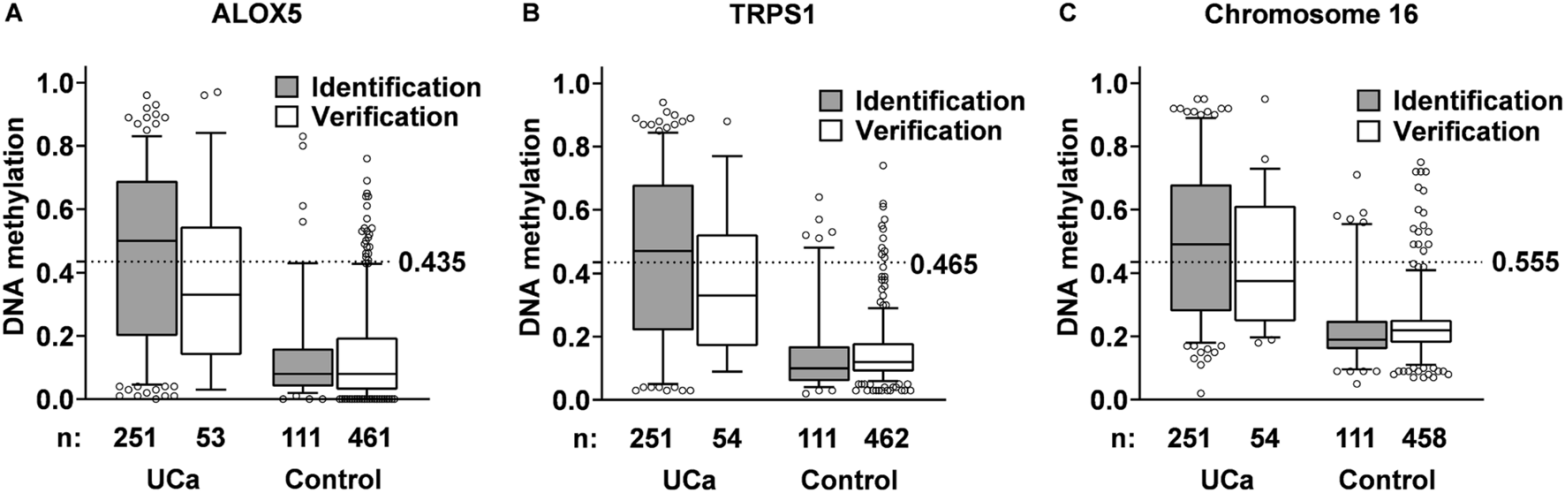
DNA-methylation in identification and verification collectives for urothelial carcinoma (UCa) and the respective controls for the male collective with < 500 leukocytes/µL urine for ALOX5 (**A**), TRPS1 (**B**) and Chromosome 16 (**C**). Values from 0 to 1 indicate 0–100% of methylated target in the respective specimens. While controls are healthy individuals or those without histologically confirmed UCa in the identification collective, the control group of the verification collective also comprises individuals with other genitourinary cancers (Table 1). Vertical bars display the median value of each group. Boxes and Whiskers indicate the 25th–75th and 5th to 95th percentile, respectively. The dashed line indicates the DNA-methylation cutoff determined in the identification collective

Comparing DNA methylation levels of the individual markers in both, the identification collective that is described in our previous work [28] and our newly collected verification collective (Table 1), we found the DNA methylation disparities between male UCa cases and controls to be notably reduced by about 15% across the analyzed targets in the “UroSpec” collective. However, we could still confirm strong and significant (p < 0.0001) differences (25, 21 and 16% median methylation difference, respectively) in urinary DNA methylation between UCa-cases and controls for ALOX5, TRPS1 and the intergenic region on Chromosome 16, respectively (Fig. 1A–C). Therefore, we applied the individual thresholds we had previously obtained at 95% specificity (43.5% methylation for ALOX5, 47% methylation for TRPS1 and 56% methylation for the intergenic region on Chromosome 16; [28], Fig. 1, Table 2) to the present study in order to calculate the diagnostic performance.

**Table 2:** Diagnostically relevant parameters for the three CpGs and two proteins of interest alone or in (“OR”)-combination

Markers alone or in combination	Threshold	Specificity overall	Sensitivity overall	AUC overall	Specificity no UCa history	Sensitivity no UCa history	AUC no UCa history
Methylation markers							
ALOX5	0.435	95.45	39.62	0.79	95.33	48.00	0.81
TRPS1	0.465	98.05	29.63	0.85	98.14	34.62	0.83
Chromosome 16	0.555	98.25	27.78	0.83	98.35	38.46	0.86
ALOX5 or TRPS1 or Chromosome 16	See above	94.25	42.59	–	94.03	53.85	–
Protein markers							
CXCL16	648.5	94.16	30.99	0.77	94.40	30.30	0.80
TGFBI	1345.9	85.84	56.34	0.77	86.73	60.61	0.79
CXCL16 or TGFBI	See above	83.88	59.16	–	84.85	63.64	–

Methylation marker values refer to the male subpopulation overall including n = 522 men with < 500 leukocytes/µL urine with and without UCa-history whereas protein marker values refer to the mixed gender collective overall including n = 1119 men and women with and without UCa-history (Table 1)

### ALOX5, TRPS1 and Chromosome 16 DNA methylation also discriminates male UCa-cases from men with other urological cancers with < 50% sensitivity at ≥ 95% specificity

**Fig. 2:**
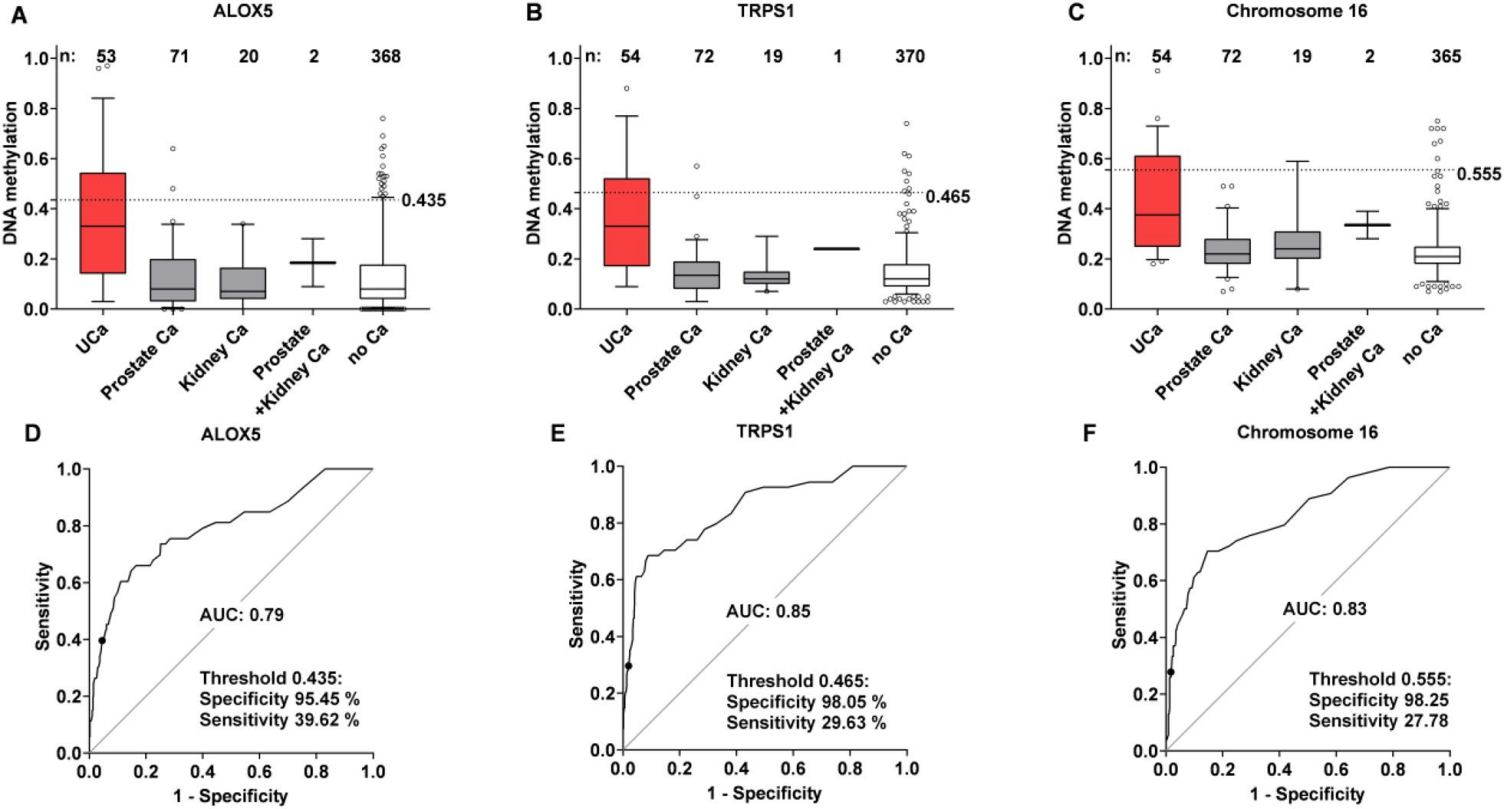
Characteristics and diagnostic performance of singular DNA-methylation markers for the male subpopulation with < 500 leukocytes/µL urine. Values from 0 to 1 indicate 0–100% of methylated target in the respective specimens. **A**–**C** DNA-methylation and diagnostic performance of singular CpGs is shown for urothelial carcinoma (UCa) patients, individuals with other urological cancers (Ca) and cancer-free individuals (no Ca) for ALOX5 (**A**), TRPS1 (**B**) and Chromosome 16 (**C**). Vertical bars display the median value of each group. Boxes and Whiskers indicate the 25–75th and 5th to 95th percentile, respectively. The dashed line marks the DNA-methylation cutoff determined in the identification collective. **D**–**F** ROC-Curves indicating the discrimination between the UCa group and a “no UCa” group also comprising other genitourinary cancers for ALOX5 (**D**), amplicon TRPS1 (**E**) and Chromosome 16 (**F**). The sensitivity and specificity at the threshold determined for each single CpG in the identification collective is indicated for each target. In addition, AUC values for individual CpGs are given

We found that the median urinary methylation of patients with prostate cancer (8% for ALOX5, 14% for TRPS1 and 22% for Chromosome 16) and kidney cancer (7% for ALOX5, 12% for TRPS1 and 24% for Chromosome 16) was similar to that of the cancer-free controls (8% for ALOX5, 12% for TRPS1 and 21% for Chromosome 16) (Fig. 2A–C). In contrast, the median methylation difference of the three investigated targets was notably higher (33–38% across the three targets) in UCa-patients with median methylation differences for ALOX5, TRPS1 and Chromosome 16 of 25%, 19% and 16% when compared to prostate cancer, and very similar or identical (26%, 21% and 16%) for the three targets when compared to kidney cancer, respectively (Fig. 2A–C). ALOX5 showed the strongest disparities, followed by TRPS1 and Chromosome 16 (Fig. 2A–C). Comprising all “no UCa” cases in one control group, individual targets displayed AUC values between 79 and 85%, Fig. 2D–F, Table 2). Sensitivities at minimum 95% specificity reached values between 28% (Chromosome 16, Fig. 2F) and 40% (ALOX5, Fig. 2D) in the overall male collective. In male patients without UCa history, the median sensitivity was 8% higher across the targets reaching up to 48% for ALOX 5 (Table 2).

### A combination of DNA methylation markers slightly increases sensitivity

To increase the diagnostic performance, we tried to apply an “or-combination” of ALOX5, TRPS1 and Chromosome 16 DNA methylation. When only one of the three markers exceeded its threshold, the sensitivity of UCa-detection increased to 43% at an only marginally reduced specificity of 94% in the male study sample. For men without a history of UCa, the “or-combination” of the three methylation markers even reached 54% sensitivity at a specificity of 94% (Table 2).

We investigated the overlap of marker positive specimen among the cancer cases where results for all three methylation markers were obtained. Among the latter, about 19% were concordantly detected by all three methylation markers (Fig. S1, left), and still 15% were positive for 2 markers. Most UCa-cases were positive for ALOX5 and among those, about 19% were exclusively detected by this marker. Vice versa, ALOX5 also accounted for 13 of 25 (52%) of the false-positive men among patients analyzed for the overlap of positive markers (Fig. S1, right). In contrast, exclusive Chromosome 16 methylation did not detect any UCa patient but accounted for three of 25 (12%) false-positive classifications in the non-UCa group (Fig. S1). However, 94% of the considered male non-UCa patients were concordantly negative for all three methylation markers (Fig. S1, right).

### Verification of urinary protein markers for UCa detection

**Fig. 3:**
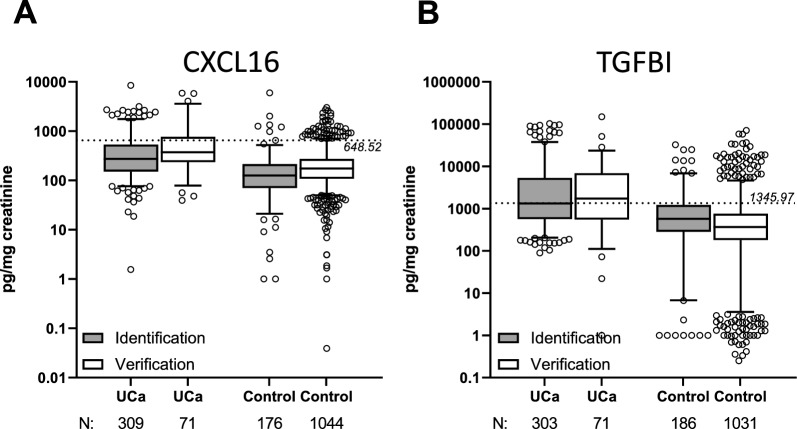
Boxplots of soluble, creatinine normalized CXCL16 and TGFBI in urine of patients with urothelial carcinoma (UCa) and in various controls in divergent patient collectives. Urinary CXCL16 concentration in UCa patients in both-the previous identification collective versus the actual verification patient collective- compared to the corresponding controls (**A**). Urinary TGFBI concentration in UCa patients in both—the previous identification collective versus the actual verification patient collective—compared to the corresponding controls (**B**). CXCL16 threshold is 648.52 pg/mg creatinine and for TGFBI 1345.97 pg/mg creatinine. N = number of analyzed patient specimens

ELISA quantification of CXCL16 and TGFBI in all collected urine samples confirmed significantly higher levels in patients with UCa (median 372.9 pg/mg creatinine for CXCL16 and 1730.2 pg/mg creatinine for TGFBI) compared to the respective controls (median 174.3 pg/mg creatinine for CXCL16 and 365.8 pg/mg creatinine for TGFBI; p < 0.0001; Fig. 3). The median values of CXCL16 (616.6 pg/mg creatinine) and TGFBI (4845.3 pg/mg creatinine) in high-grade UCa patients were significantly higher compared to those with low grade UCa (279.6 pg/mg creatinine for CXCL16 and 1083.2 pg/mg creatinine for TGFBI; Table S1) and controls (174.3 pg/mg creatinine for CXCL16 and 365.8 pg/mg creatinine for TGFBI; p < 0.0001). Within the group of UCa patients without UCa history (= primary UCa) we observed increased median concentrations of CXL16 (504.0 pg/mg creatinine) in comparison to those controls with and without a history of UCa (372.9 pg/mg creatinine; Fig S5) and controls without UCa history (172.2 pg/mg creatinine). In the case of TGFBI, the UCa history was irrelevant for the median urinary biomarker concentration in the UCa patient group (1744.8 pg/mg creatinine in primary UCa patients vs. 1730.2 pg/mg creatinine UCa patients comprising those with and without UCa history; Fig. S5). However, we observed a distinct higher median concentration of TGFBI in the low grade primary UCa group (3411.1 pg/mg creatinine) vs. the low grade UCa group (1083.2 pg/mg creatinine), but no difference in the patients with high grade UCa, respectively (4817.1 pg/mg creatinine TGFBI in primary high grade UCa vs. 4845.3 pg/mg creatinine in high grade UCa patients with and without history; Fig. S5).

**Fig. 4:**
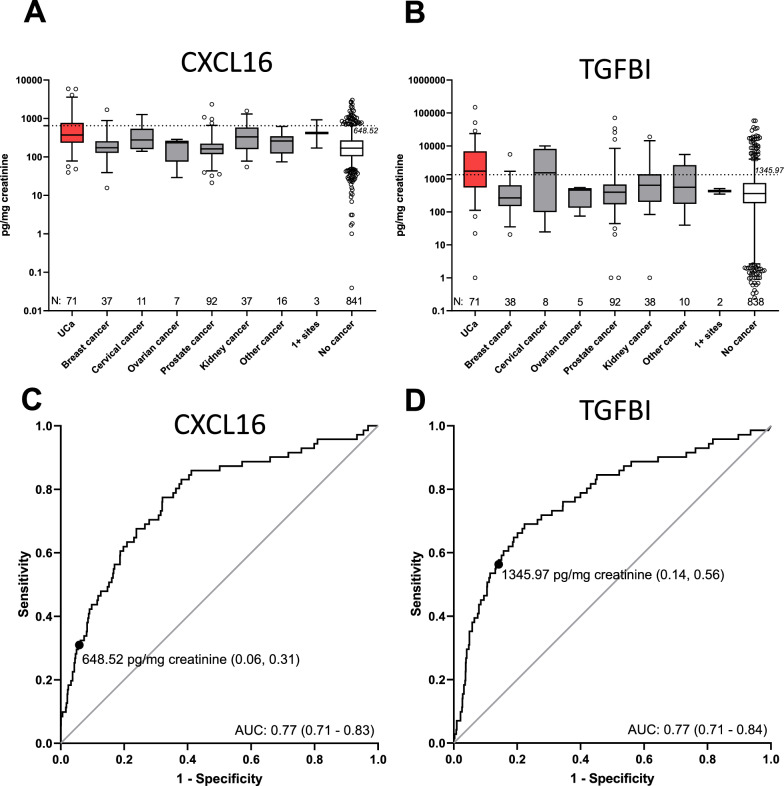
Boxplots of soluble, creatinine normalized CXCL16 and TGFBI in urine of patients with urothelial carcinoma (UCa) or other tumors and those patients without cancer. Urinary concentration of CXCL16 (**A**) and TGFBI (**B**) in UCa patients (red) in comparison to patients with other primary tumors and those having no cancer. ROC analysis of creatinine normalized CXCL16 (**C**) and TGFBI (**D**) in urine of UCa patients in comparison to patients with other cancers and the control group. N = number of analyzed patient specimens

In the next step we investigated the urinary biomarker concentration in detail as well as the specificity and sensitivity within the whole patient collective. In patients with UCa the median urinary concentration of CXCL16 was distinctly higher than in other cancers, whereas we observed a slight increase of CXCL16 in the urine of kidney cancer patients, too (Fig. 4A). However, considering solely the primary UCa cases, we can clearly separate the UCa patients (median 504.1 pg/mg creatinine) from those patients having kidney cancer (median 328.1 pg/mg creatinine; Fig. 4A). Regarding TGFBI, we found the highest median concentration in the urine of UCa patients independent from UCa history (1730.2 pg/mg creatinine), and an enhanced median concentration in the group of women with cervical cancer (N = 8; 1530.9 pg/mg creatinine), but no noticeable values in the group of kidney cancer patients (643.0 pg/mg creatinine; Fig. 4B).

For the discrimination of UCa cases from other cancer patients and controls (independent from UCa history), the sensitivities and specificities of CXCL16 based on the 95th percentile of CXCL16 in hospital controls from our previous study (648.5 pg/mg creatinine) were calculated. ROC curve analysis of creatinine—normalized CXCL16 and TGFBI concentrations in urine are presented in Fig. 4C, D. Thereby, UCa vs. other tumors and controls revealed a sensitivity of 31% and a specificity of 94% (Fig. 4C), and a sensitivity of 30% and a specificity of 94% in cases without UCa history, respectively (Fig. S6C; Table 2). For the comparison of all UCa patients *vs*. those with other cancers and no cancer at all, we obtained an AUC of 0.77 (95% CI 0.71–0.83; Fig. 4C) for CXCL16 and an AUC of 0.77 (95% CI 0.71–0.84; Fig. 4D) for TGFBI. Using the 95th percentile of TGFBI in hospital controls from our previous study as cut-off (1346.0 pg/mg creatinine), a sensitivity of 56% and a specificity of 85% was obtained (Table 2; Fig. 4). Separate ROC analysis considering only patients without history of UCa in general resulted in a slight improvement of AUC values in primary UCa patients compared to patients with other cancer or control patients without cancer (Fig. S6D).

### Combination of urinary protein markers CXCL16 and TGFBI

To assess the benefit of combining the analysis of urinary CXCL16 and TGFBI, we calculated Venn-Diagrams for the overlap of positive protein biomarkers within all urines analyzed (Fig. S7A). A biomarker was considered positive when the concentration was higher than the threshold of 648.5 pg/mg creatinine for CXCL16 and 1346.0 pg/mg creatinine for TGFBI, respectively (Table 2). From the 71 UCa cases with a history of UCa, 59% were positive for CXCL16 and/or TGFBI and 28% UCa cases were detected by both markers. 41% UCa patients were false-negative and remained undetected by both markers. In comparison and among patients with obtained values for both markers within the non-UCa group, 864 of 1028 patients were truly negative, 16% of patients were detected as false-positive, and 4% were false-positive for both markers. Also, 10% had high urinary TGFBI concentrations although having no UCa (Fig. S7A). Considering only patients without UCa history did not change the number of truly positive UCa patients detected by both markers (27%) or the proportion of false-positive patients (15%; Fig. S7B) either.

### Or-combination of urinary methylation and protein biomarkers strongly increases the sensitivity of UCa diagnosis at the cost of a reduced specificity

**Fig. 5:**
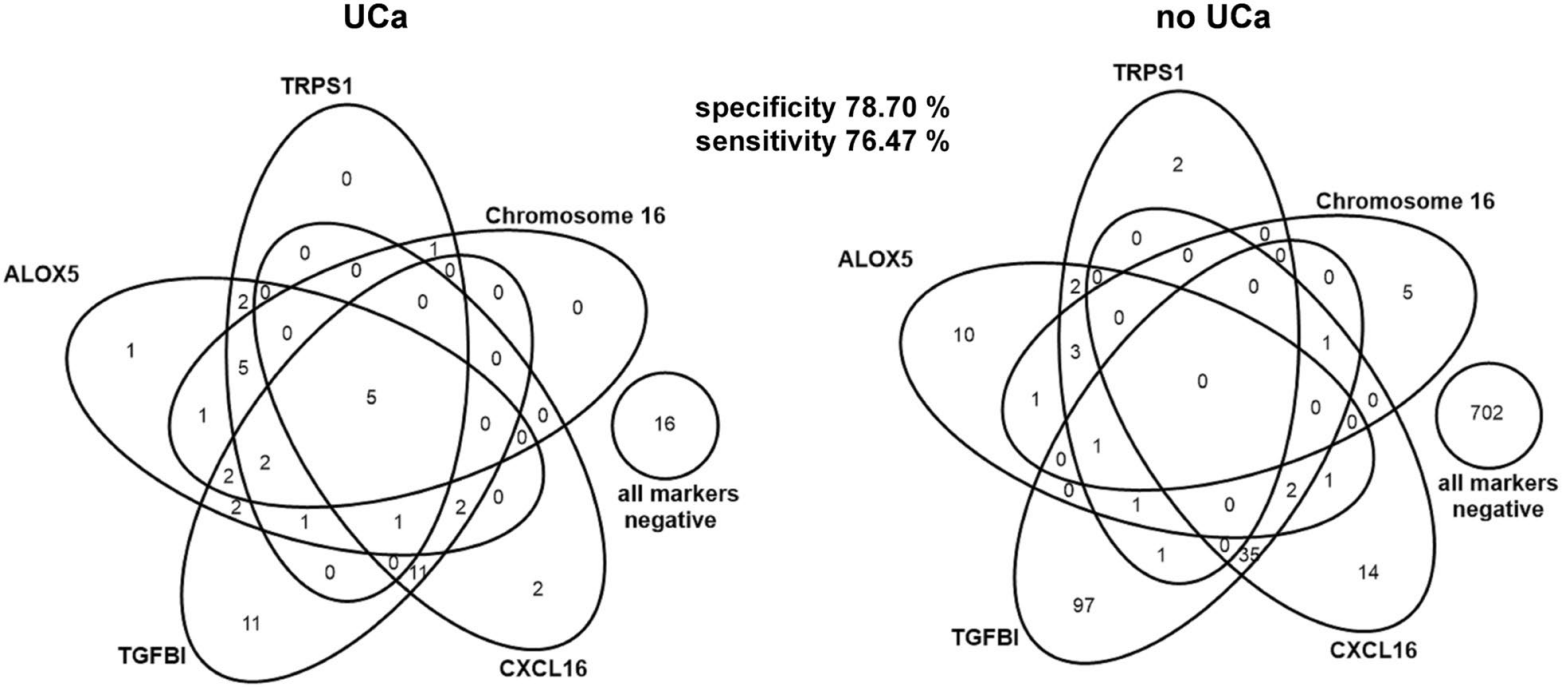
Venn-diagrams for overlap of positive (biomarker ≥ threshold) methylation and protein biomarkers in UCa patients and UCa-free participants (no UCa). CXCL16 threshold is 648.52 pg/mg creatinine. TGFBI threshold is 1345.97 pg/mg creatinine. ALOX5 threshold is 43.5% methylation, TRPS1 threshold is 46.5% methylation, and Chromosome 16 threshold is 55.5% methylation. Overall, 71 UCa cases and 1048 non-UCa patients were investigated. 6 UCa patients (one positive for TRPS1, one positive for Chromosome 16, one positive for CXCL16 and three positive for TGFBI) and 170 non-UCa patients (one positive for TRPS1, one positive for Chromosome 16, eight positive for CXCL16 and seven positive for TGFBI) with at least one missing value are not shown

First, we applied Venn-Diagrams to visualize the overlap of positive markers in UCa patients (Fig. 5; N = 65) and in non-UCa patients (Fig. 5; N = 878). Overall, 75% of the UCa patients represented by the diagram were detected by at least one positive biomarker (proteins: CXCL16, TGFBI; DNA methylation: ALOX5, TRPS1, Chromosome 16), but only 5 UCa patients (8%) were detected by all five biomarkers at the same time. Among the UCa cases, 37% were recognized exclusively by protein markers (with most cases being detected by TGFBI) and 15% exclusively by DNA methylation markers (with most cases being detected by ALOX5). 25% of UCa cases were false negative and not recognized by any of the five markers at all (Fig. 5). In the non-UCa group, 80% of patients were truly negative. According to the high number of cases detected by TGFBI, CXCL16 and ALOX5, these markers are responsible for the increased number of false-positive patients (20%) in the non-UCa group.

Next, we checked various statistical combination methods. For the OR-combination we used the known thresholds for all five biomarkers from the identification studies, and a patient was classified as positive if at least one biomarker exceeded its threshold from the identification study (Table 3). For example, if positivity of either CXCL16 (cutoff: 648.5 pg/mg creatinine) or ALOX5 (cutoff: 44% methylation) or any other marker (cutoffs see Table 2) is sufficient for a positive test result, the combination of all protein and DNA methylation markers reaches a sensitivity of 76.4% and a specificity of 79% (Table 3; N = 1119). The sensitivity is further increased to 84% with a specificity of 80% when analysis was performed only with patients who never had UCa before (N = 1019, Fig. S8, Table 3). In contrast to our previous observations with the methylation markers, when combining all markers (protein and DNA methylation) the identification of patients with UCa is independent from gender (Table 3).

**Table 3:** Results of biomarker combination analysis for the classification of UCa for the different collectives analyzed

Collective	N	Method	N used for performance	Specificity*	Sensitivity
Men and women	1119	OR	960	78.70%	76.47%
	1119	PanelomiX	943	97.3% (96.7%)	72.3% (58.2%)
Men and women without history of UCa	1019	OR	871	79.88%	83.87%
	1019	PanelomiX	858	98.1% (97.5%)	79.3% (56.6%)
Men	653	OR	552	76.83%	78.33%
	653	PanelomiX	541	97.5% (95.6%)	66.7% (55.5%)
Men without history of UCa	580	OR	486	77.85%	83.33%
	580	PanelomiX	476	97.1% (95.9%)	75.0% (52.4%)

The OR-combination is positive if at least one marker exceeds its threshold value and negative if all markers are negative. Patients were excluded if there were missing marker values and the remaining ones were negative. For PanelomiX, the results for 10 × 3-CV (cross-validation) are given in brackets. *PanelomiX with constraint on specificity: at least 95%

As an alternative combinatory approach, we used the iterative threshold-based biomarker and score combining algorithm “PanelomiX” to identify the best biomarker panels for the classification of UCa with a fixed specificity of at least 95%. With PanelomiX, combining all five markers resulted in a sensitivity of 72% with a specificity of 97% (Table 3; N = 1119). The 10 × 3 cross-validation yielded a mean sensitivity of 58% with a mean specificity of 97%. Excluding patients with UCa history, analysis reached a sensitivity of 79% with a specificity of 98% (N = 1019) independent from gender (10 × 3 cross-validation: mean sensitivity 57%, mean specificity 97%).

## Discussion

According to current urological guidelines, cystoscopy is still the standard of care to diagnose primary and recurrent UCa, but is invasive, cost-intensive, and often causes pain and bleeding, especially in male patients [6, 35, 36]. Therefore, numerous approaches have been undertaken to identify non-invasive urinary biomarkers at different molecular levels (protein, RNA, DNA) and in different urinary matrices (cell pellet, supernatant, exosomes) for UCa-detection [37–39], and our group has contributed to this wealth by identifying urinary UCa-biomarkers at the level of protein analysis and DNA-methylation [25–27, 40].

As an impairment of our own and other’s previous identification and verification studies, UCa is usually distinguished from healthy controls or — at best — from patients experiencing benign urological problems [25–27], but not from patients with neoplasms of other tissues of the genitourinary tract (e.g., prostate, kidney, cervix) that might also shed cells into the urine, especially at advanced stages [20, 29]. Therefore, in the present study we are among the first to evaluate the UCa-specificity of our previously identified markers in a heterogeneous collective of genitourinary patients including cancers and the whole spectrum of benign disorders affecting the respective tissues in both genders. So far, no single biomarker has sufficient predictive power to be implemented in clinical management as screening marker for UCa diagnosis in the general population. Accordingly, numerous panels have been described in the literature, usually combining the analyses of different targets assessed with the same method and identical matrices, separately addressing low-grade and high-grade disease or recurrence. This appears the most promising way forward to improve risk stratification before transurethral resection of the bladder and may specifically help to detect high-grade tumors. Especially in high-risk patients, extremely sensitive screening tools are required not to miss disease progression, tumor recurrence and persistence (e.g., pTis), as this disease can be fatal if detection fails. With this in mind, we have outlined the analytical validation of every single biomarker alone and in combination. As a second novelty we analyzed whether biomarkers that were assessed by different methods and in different urinary fractions (supernatant and cell pellet) of the same urinary specimen can contribute to a refined UCa detection by applying different statistical approaches. Integrating these novel aspects, we hope to contribute to the reduction of necessary cystoscopies by a marker-based selection of high-risk patients.

Regarding the individual markers identified in our previous identification studies, the current UroSpec collective served as an independent verification collective. By using the thresholds obtained in our previous studies, we could confirm substantial differences in both DNA methylation and protein markers also for the heterogeneous new collective when comparing UCa cases and the heterogeneous group of UCa-free individuals recruited in urological and gynecological physicians’ offices (Figs. 1 and 3).

### Marker differences between UCa cases and non-UCa controls are confirmed in the verification collective UroSpec

Regarding DNA-methylation markers, lower median methylation values were observed for male UCa patients in the identification collective when compared to the UroSpec verification collective, which is in line with a reduced sensitivity in the UroSpec study sample (Fig. 1, Table 2). This might in part be due to the (by 10%) higher fraction of individuals with a history of UCa in the collective analyzed here when compared to the identification study, as — concordant with other markers at multiple molecular levels described in the literature and with our own previous results [25–28, 41] — individuals diagnosed with primary UCa could be identified with a notably higher sensitivity than those under surveillance for recurrent tumors, presumably to a larger size of the tumors and thus a higher chance of shedding cells or releasing soluble protein components into urine when compared to patients under regular inspection (Table 2).

### Median urinary DNA methylation is similar in cancer-free individuals and men with prostate or kidney cancer

Interestingly, all three methylation markers investigated displayed strong median methylation differences not only when compared to cancer-free controls, but also in comparison with prostate and kidney cancer patients. In a previous study, we analyzed the methylation status of our three markers and found ALOX5 to be highly methylated in 2/3 prostate and 1/3 kidney cancer cell lines [40]. In the present investigation, the vast majority of around 70 prostate and around 20 kidney cancer urinary specimens fell below the individual threshold for UCa-detection. The mismatch among these two studies suggests that the cell-culture-based findings might not be universally valid as each cell line represents one single individual only. Another interpretation of this discrepancy might be that although the presence of prostate and kidney cells in urine has been described before [20, 29], the latter is of minor relevance for UCa detection from the cell pellet due to a small fraction of the respective cells (at least in male urine specimen with low leukocyte counts) even in advanced stages of other male urogenital cancers.

### Gender differences of urinary UCa detection by methylation markers cannot conclusively be studied in the present collective

In contrast to our previous results, we could not confirm gender differences (Figs. 1, 2, Figs. S2, S3, Table 2, Table S2). However, as the UroSpec collective only included seven female UCa cases thus not allowing for a generalized conclusion with respect to gender (see supplementary material). Nevertheless, because in previous studies (and also in the current collective though in a small number of cases), we observed only few false-positive UCa findings in women [27].

### ALOX5 is confirmed to be the most informative DNA methylation marker for UCa

ROC- and overlap-analyses confirmed ALOX5 as the most meaningful individual methylation marker whereas Chromosome 16 alone did not contribute to the detection of UCa cases but yielded several false-positive findings and therefore turned out to be of no additional diagnostic value when applying an OR-combination (Fig. 2, Table 2, Fig. S1). However, inclusion of Chromosome 16 into the PanelomiX approach further improved UCa-detection as different thresholds were used.

### Protein markers

In this verification study ELISA quantification of CXCL16 and TGFBI confirmed significantly higher levels in patients with UCa (median 372.9 pg/mg creatinine for CXCL16 and 1730.2 pg/mg creatinine for TGFBI) compared to the respective controls (median 174.3 pg/mg creatinine for CXCL16 and 365.8 pg/mg creatinine for TGFBI). This is in line with the results of our identification collective wherein the median concentration of urinary CXCL16 was 274.3 pg/mg creatinine in UCa in comparison to 126.2 pg/mg creatinine in the control group, and for TGFBI 1321.0 pg/mg creatinine in the UCa group vs. 574.0 pg/mg creatinine in the controls, respectively. Thus, we can confirm both urinary biomarkers as potential tools for diagnosing high-grade UCa independent from gender and the presence of other cancer-disease.

With regard to CXCL16, we also observed a slight increase of CXCL16 in the urine of kidney cancer patients when compared to non-cancer patients. However, considering solely the primary UCa cases we can clearly separate UCa patients from those patients having kidney cancer (Fig. 4). Regarding TGFBI, we found the highest median concentration in the urine of UCa patients independent from UCa history, and an enhanced median concentration in the group of women with cervical cancer when compared to non-cancer patients. However, the UroSpec collective included only eight cervical cancer cases which limits the interpretation of this observation. Future studies are warranted to confirm these findings, because in another study TGFBI expression was also shown to be increased in cervical cancer tissue [42].

With regard to specificity and sensitivity of both protein markers, the results were similar as in the identification study, and combining both marker results did not further improve the detection of UCa.

### Combination of biomarkers can increase sensitivity at high specificity

As expected with the OR-combination of highly specific single markers, (i.e., when at least one marker needs to exceed its threshold to classify the respective individual as UCa-positive), the sensitivity is increased whereas a decrease in specificity is observed. This was observed for markers at both levels (DNA and protein) and in both investigated sub-collectives (Table 2).

As already previously discussed in our DNA methylation-studies [27, 28], the calculated sensitivities and specificities are difficult to compare to other studies for several reasons: first, the size and composition of the study population including the characteristics of the cases and control groups (including e.g. age, gender, grading and the history of UCa, urinary blood cell count) as well as the ratio of cases and controls significantly influence the marker performance. In addition, in order to reduce the number of false-positive findings, we used a priori defined high specificity in our methylation marker identification studies. Such an approach is rarely performed by other researchers who mostly balance sensitivity and specificity. In contrast, a ‘balanced’ approach has been used for our protein markers. Therefore, the performances of the molecular levels (DNA methylation and protein) cannot directly be compared in our study.

A recent review dealing with DNA methylation markers described the five most promising markers with sensitivities ranging from 61 to 87% and a specificity of 89–97% [19]. However, this review did not discuss relevant factors influencing results. In 2023, the first urine biomarker methylation test Bladder EpiCheck® received FDA clearance for monitoring of non-muscle invasive bladder cancer (NMIBC) recurrence in conjunction with cytology [23]. Several studies analyzing the performance of Bladder EpiCheck® test (using 15 methylation markers) showed a sensitivity of 90% and a specificity of 83% in patients with NMIBC under surveillance [22, 23], but only when excluding low grade carcinoma. Altogether, several individual methylation markers and panels gained attention in prospective studies, but the lack of strong evidence, the inconsistency in proof and validation of performance still limits their use in daily clinical care.

Our protein biomarkers CXCL16 and TGFBI, used in combination can discriminate UCa cases with a sensitivity of 31% and 56% and a specificity of 94% and 85%, respectively. There are numerous other test assays measuring single biomarkers or panels in patient urine with high clinical potential. For example, the ADXBLADDER urine test detects the MCM5 protein in urine sediment and three studies comprising more than 2000 patients resulted in a pooled sensitivity and specificity of 71% and 76%, respectively [43]. Based on a retrospective multi-marker study including patients with primary and recurrent BC, the quantitative POC assay UBC® Rapid assay targeting cytokeratin 18 and 20, showed a sensitivity of 46.4% and a specificity of 75% for low grade BC, and 70.5% and 75.5% for high-grade BC, respectively. However, combining its use with BC risk factors including age, smoking status and haematuria increased its impact as tool for screening patients for primary low-grade BC and especially primary high-grade BC [44].

Here, we extended analysis by combining all methylation and protein markers, up to five in total, yielding a convincingly high specificity of 97% at a sensitivity of 72% for the identification of UCa patients within a heterogeneous collective of cancer-free individuals or patients suffering from urological or gynecological cancers**.**

Fixing the specificity of the combination to at least 95% in the PanelomiX-approach, the sensitivity reached 72%, exceeding the highest value obtained individually or in the “OR-combination” by 16% in the overall collective and by even 20% in the men and women excluding individuals with UCa-history, which almost equals the sensitivity of the OR-combination algorithm (Table 3). However, these results are not directly comparable to the OR-combination as the thresholds in PanelomiX were not selected from independent data. Therefore, the cross-validated mean performances from PanelomiX show a decreased sensitivity, although the specificity remained at a high level (Table 3). Unfortunately, the platform offering a web-based PanelomiX analysis is not further supported and while the R-package “PanelomiX” is still available via GitHub, the implementation is highly experimental but promising.

In summary, our biomarkers deliver reliable results, confirming the targets identified in our previous studies. They might, together with other factors such as gender, age and smoking status, provide data for decision making in unclear cases, i.e. whether or not an invasive cystoscopy is needed in patients with minor urinary symptoms such as (repeated) microhaematuria. Like all molecular biological and biochemical analyses, our test assays are time-consuming and cost intense. However, considering the costs for the gold standard (including cystoscopy equipment and maintenance, staff time) and its invasive nature, these disadvantages might be outweighed by the benefits of early detection sparing unnecessary cystoscopies and — consequently — reduce discomfort, bleeding and anxiety of the patients [45].

Strengths of our study include the large sample size of more than 1000 urine samples collected under real-life conditions at five different clinics, thus representing a heterogenous study group for verification of our markers. A limitation of our study is that we did not collect follow-up data. Moreover, not every individual with urinary symptoms received a cystoscopy. Thus, we were unable to correlate the false-positive findings with incident UCa cases or subsequent recurrent cancers. Conclusions regarding gender-specific results are difficult, because they are based on seven female UCa cases. Further, grading information for the majority of UCa cases was missing. For further confirmation of our results more UCa cases with information on grading and a higher number of female patients with UCa are needed in the future.

## Conclusion

In summary, we confirmed our previously identified methylation biomarkers ALOX5, TRPS1 and Chromosome 16 as well as our protein biomarkers CXCL16 and TGFBI to discriminate UCa from cancer-free controls in a large and independent collective. In addition, we also examined the tissue specificity of our urinary markers and showed in a large study collective with over 1000 patients that our previously identified protein and DNA methylation biomarkers can well distinguish UCa from frequent non-UCa urological (prostate and kidney cancer) and gynecological (breast, ovarian, cervix, uterus, and vaginal) cancers. For our collective, the combination approach yielded a high specificity at a diagnostically useful sensitivity for the identification of UCa patients among a heterogeneous collective of cancer-free individuals and patients suffering from urological or gynecological non-UCa cancers and thus might help to reduce the number of unnecessary cystoscopies, especially in patients without a history of UCa. In general, combining markers targeting different targets and different types of biomolecules into a panel might also be an option for other diseases and molecular levels of interest. A rising sensitivity might justify higher analysis costs.

## Supplementary Information

Supplementary Material 1.

Supplementary Material 2.

## Data Availability

The datasets analyzed for the current study are available from the corresponding author on reasonable request.

## Abbreviations

ALOX5: Referred to as Amplicon02_CpG6 (with the C of interest in position Chr10:45,418,979 according to Genome build GRCh38/hg38)
AUC: Area under curve
BC: Bladder cancer
BTA: Bladder tumor antigen
CIS: Carcinoma in situ
CpG: Cytosines followed by guanine residues
CXCL16: C-X-C Motif Chemokine Ligand 16
DNA: Desoxyribonucleic acid
ELISA: Enzyme-linked immunosorbent assay
FDA: U.S. Food and Drug Administration
IPA: Institute for Prevention and Occupational Medicine of the German Social Accident Insurance, Institute of the Ruhr University Bochum
Min: Minutes
NMIBC: Non-muscle invasive bladder cancer
PBS: Phosphate buffered saline
PCR: Polymerase chain reaction
RNA: Ribonucleic acid
ROC: Receiver operating characteristic
TGFBI: Transforming growth factor beta induced protein
TRPS1: Is referred to Amplicon 35_CpG7 (with the C of interest in position Chr8:115,667,708 according to Genome build GRCh38/hg38)
UCa: Urothelial carcinoma
