# Supplementary material for: Urinary DNA-methylation and protein biomarkers identify urothelial carcinoma among other genitourinary diseases and cancer-free individuals
Source: J Transl Med. 2024 Nov 26;22:1061. doi: 10.1186/s12967-024-05844-x (PMC11590282; doi:10.1186/s12967-024-05844-x)
Supplement: Supplementary file 1 — Supplementary Material 1. [file 12967_2024_5844_MOESM1_ESM.pptx]

## Slide 1
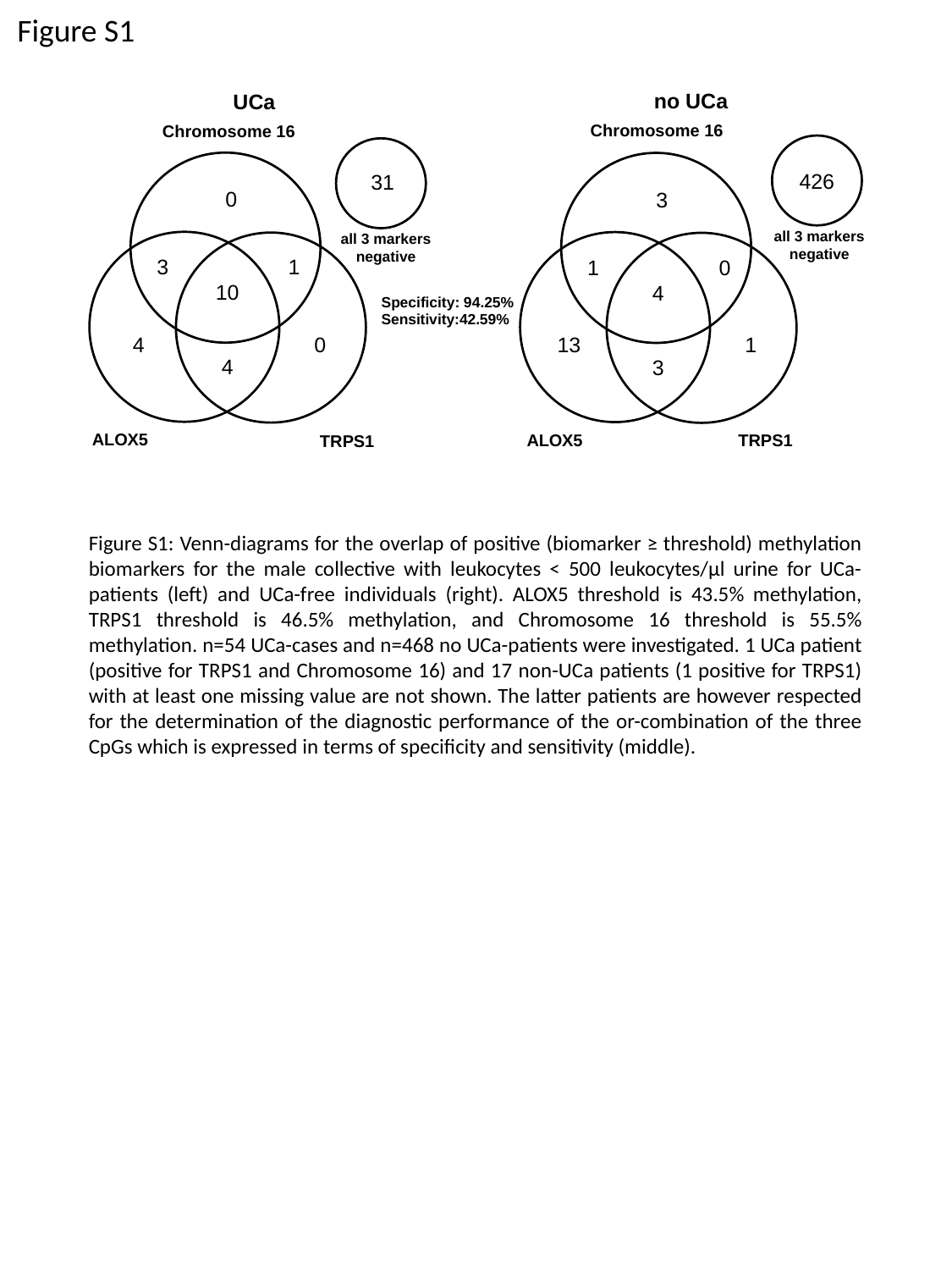

Figure S1
Figure S1: Venn-diagrams for the overlap of positive (biomarker ≥ threshold) methylation biomarkers for the male collective with leukocytes < 500 leukocytes/µl urine for UCa-patients (left) and UCa-free individuals (right). ALOX5 threshold is 43.5% methylation, TRPS1 threshold is 46.5% methylation, and Chromosome 16 threshold is 55.5% methylation. n=54 UCa-cases and n=468 no UCa-patients were investigated. 1 UCa patient (positive for TRPS1 and Chromosome 16) and 17 non-UCa patients (1 positive for TRPS1) with at least one missing value are not shown. The latter patients are however respected for the determination of the diagnostic performance of the or-combination of the three CpGs which is expressed in terms of specificity and sensitivity (middle).

## Slide 2
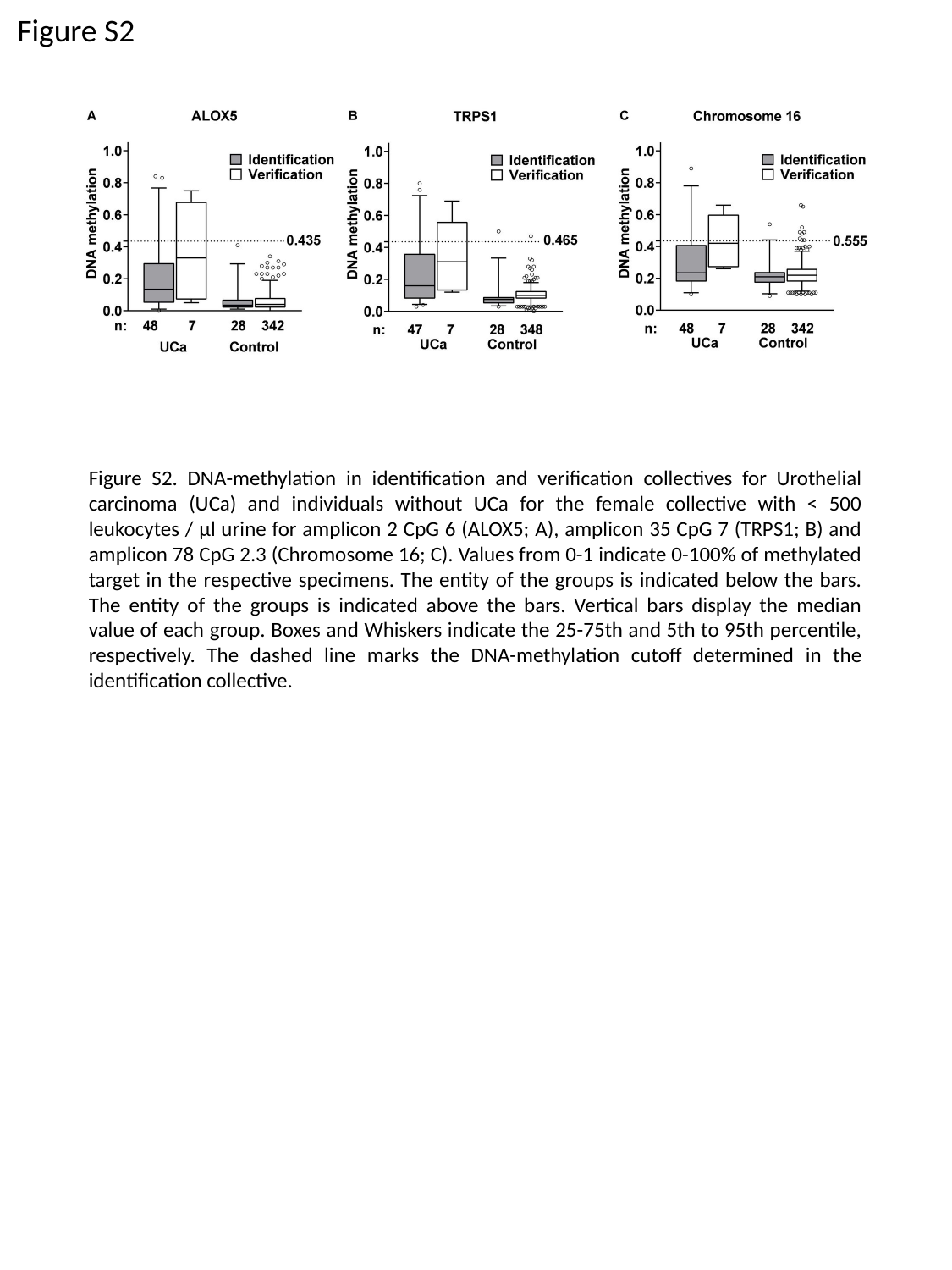

Figure S2
Figure S2. DNA-methylation in identification and verification collectives for Urothelial carcinoma (UCa) and individuals without UCa for the female collective with < 500 leukocytes / µl urine for amplicon 2 CpG 6 (ALOX5; A), amplicon 35 CpG 7 (TRPS1; B) and amplicon 78 CpG 2.3 (Chromosome 16; C). Values from 0-1 indicate 0-100% of methylated target in the respective specimens. The entity of the groups is indicated below the bars. The entity of the groups is indicated above the bars. Vertical bars display the median value of each group. Boxes and Whiskers indicate the 25-75th and 5th to 95th percentile, respectively. The dashed line marks the DNA-methylation cutoff determined in the identification collective.

## Slide 3
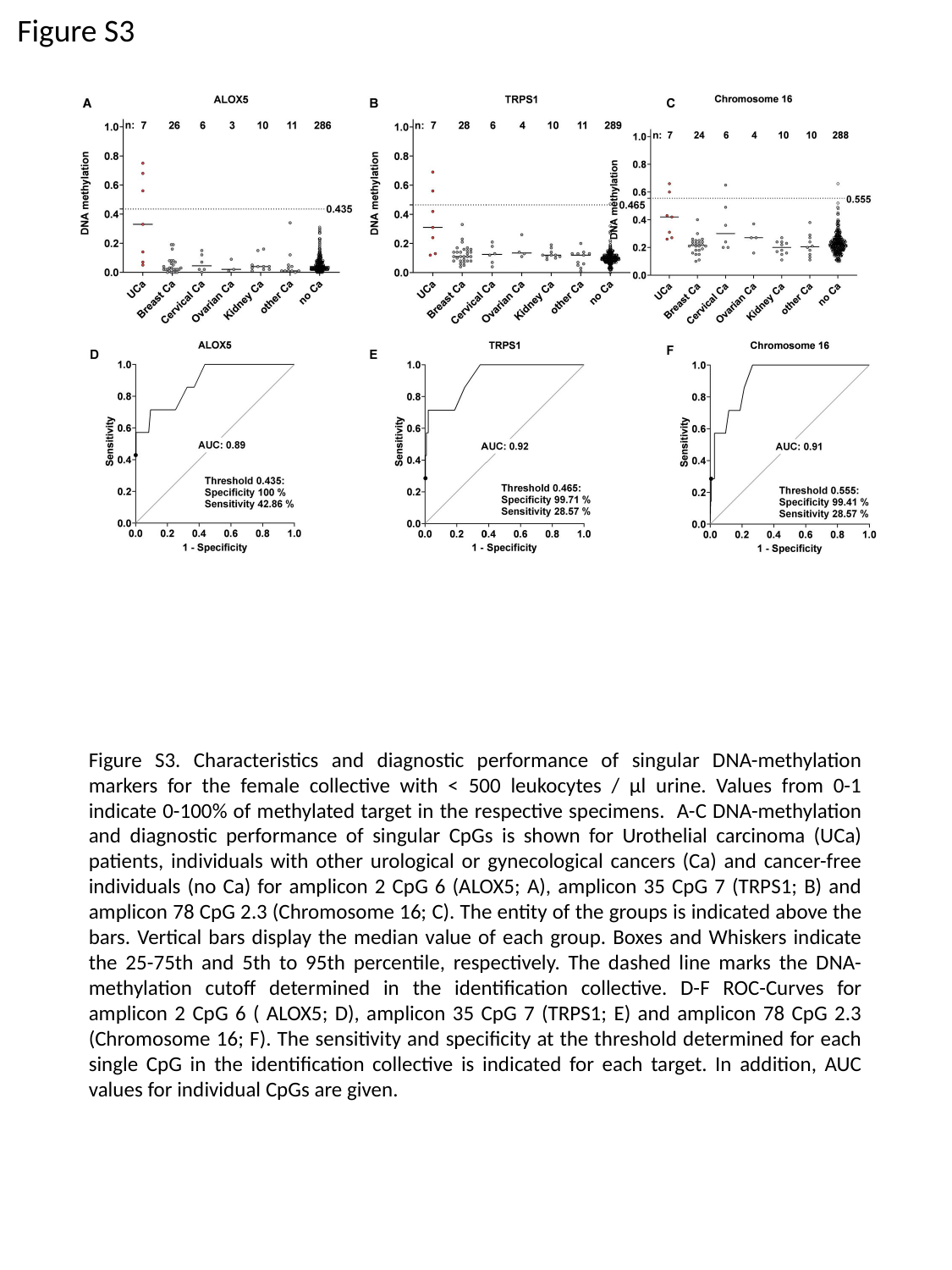

Figure S3
Figure S3. Characteristics and diagnostic performance of singular DNA-methylation markers for the female collective with < 500 leukocytes / µl urine. Values from 0-1 indicate 0-100% of methylated target in the respective specimens. A-C DNA-methylation and diagnostic performance of singular CpGs is shown for Urothelial carcinoma (UCa) patients, individuals with other urological or gynecological cancers (Ca) and cancer-free individuals (no Ca) for amplicon 2 CpG 6 (ALOX5; A), amplicon 35 CpG 7 (TRPS1; B) and amplicon 78 CpG 2.3 (Chromosome 16; C). The entity of the groups is indicated above the bars. Vertical bars display the median value of each group. Boxes and Whiskers indicate the 25-75th and 5th to 95th percentile, respectively. The dashed line marks the DNA-methylation cutoff determined in the identification collective. D-F ROC-Curves for amplicon 2 CpG 6 ( ALOX5; D), amplicon 35 CpG 7 (TRPS1; E) and amplicon 78 CpG 2.3 (Chromosome 16; F). The sensitivity and specificity at the threshold determined for each single CpG in the identification collective is indicated for each target. In addition, AUC values for individual CpGs are given.

## Slide 4
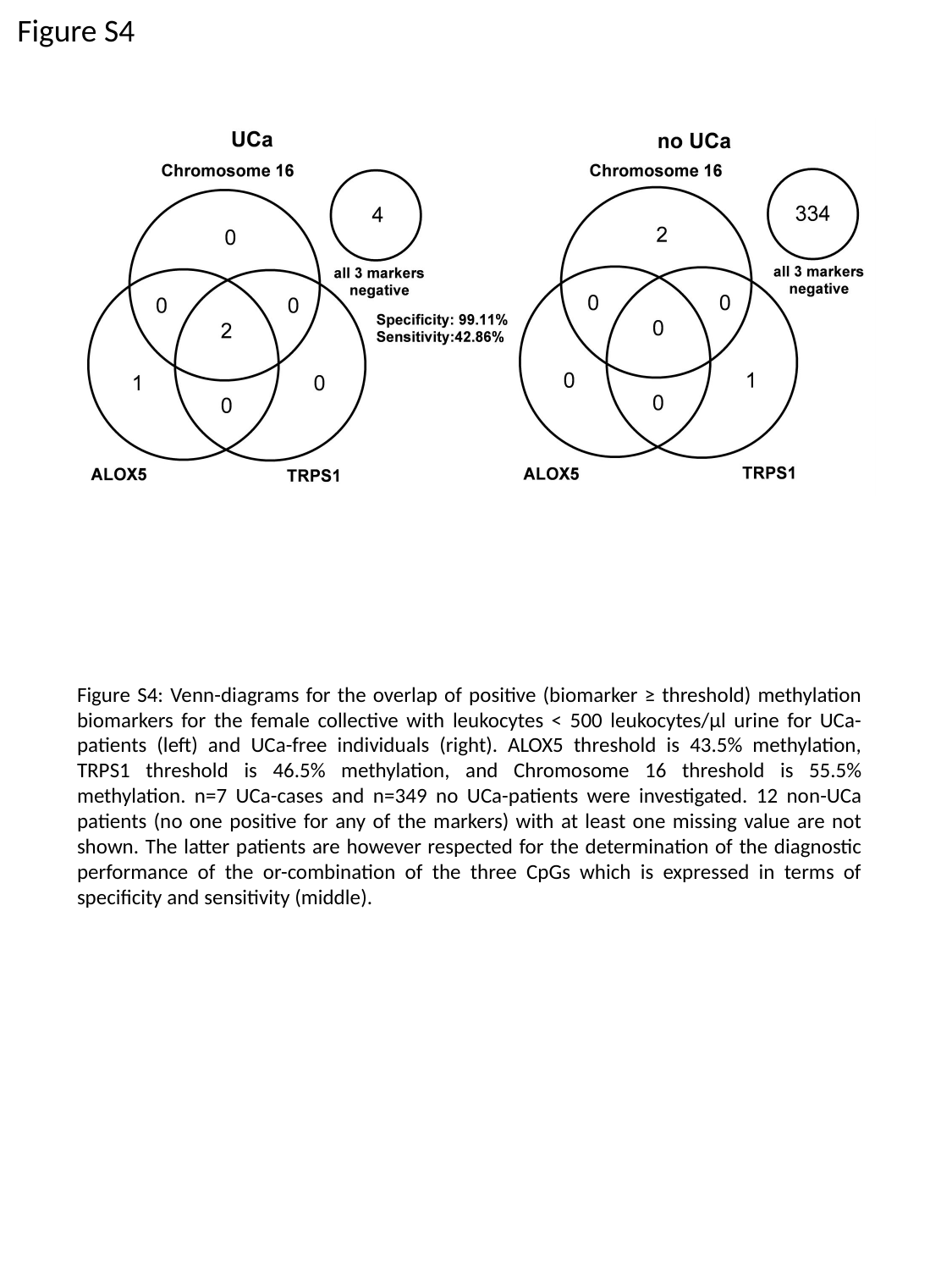

Figure S4
Figure S4: Venn-diagrams for the overlap of positive (biomarker ≥ threshold) methylation biomarkers for the female collective with leukocytes < 500 leukocytes/µl urine for UCa-patients (left) and UCa-free individuals (right). ALOX5 threshold is 43.5% methylation, TRPS1 threshold is 46.5% methylation, and Chromosome 16 threshold is 55.5% methylation. n=7 UCa-cases and n=349 no UCa-patients were investigated. 12 non-UCa patients (no one positive for any of the markers) with at least one missing value are not shown. The latter patients are however respected for the determination of the diagnostic performance of the or-combination of the three CpGs which is expressed in terms of specificity and sensitivity (middle).

## Slide 5
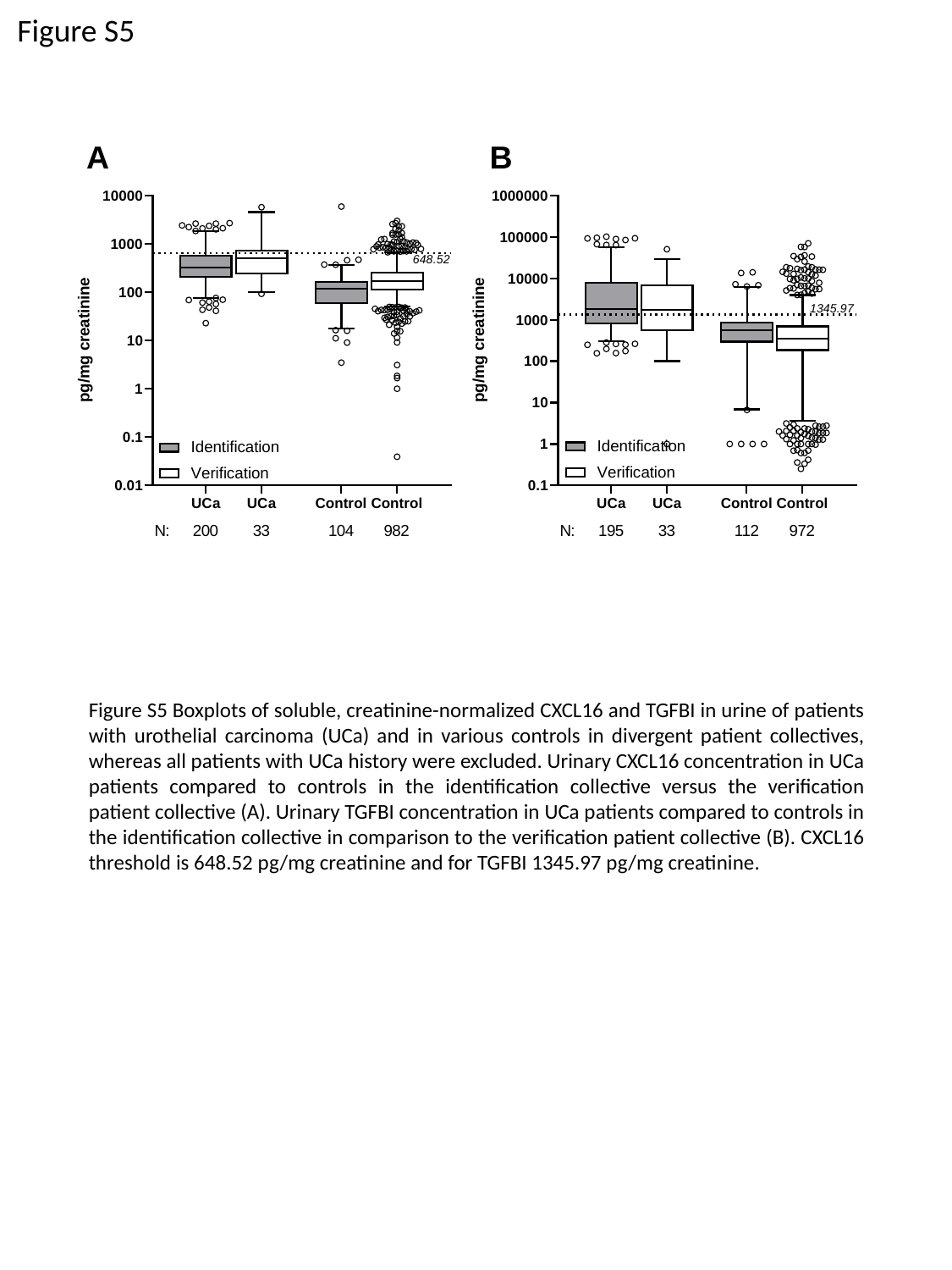

Figure S5
A
B
Figure S5 Boxplots of soluble, creatinine-normalized CXCL16 and TGFBI in urine of patients with urothelial carcinoma (UCa) and in various controls in divergent patient collectives, whereas all patients with UCa history were excluded. Urinary CXCL16 concentration in UCa patients compared to controls in the identification collective versus the verification patient collective (A). Urinary TGFBI concentration in UCa patients compared to controls in the identification collective in comparison to the verification patient collective (B). CXCL16 threshold is 648.52 pg/mg creatinine and for TGFBI 1345.97 pg/mg creatinine.

## Slide 6
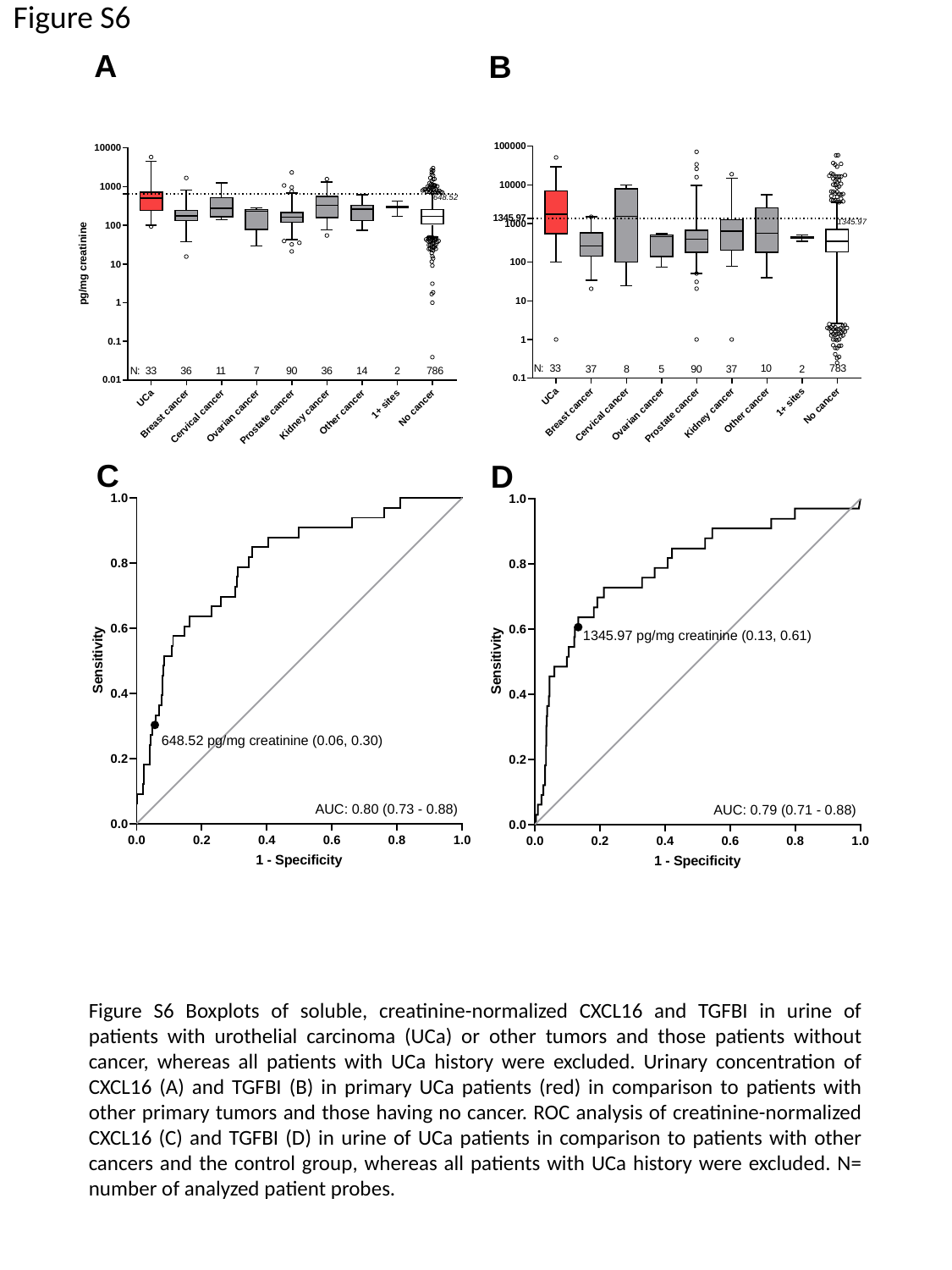

Figure S6
A
B
C
D
Figure S6 Boxplots of soluble, creatinine-normalized CXCL16 and TGFBI in urine of patients with urothelial carcinoma (UCa) or other tumors and those patients without cancer, whereas all patients with UCa history were excluded. Urinary concentration of CXCL16 (A) and TGFBI (B) in primary UCa patients (red) in comparison to patients with other primary tumors and those having no cancer. ROC analysis of creatinine-normalized CXCL16 (C) and TGFBI (D) in urine of UCa patients in comparison to patients with other cancers and the control group, whereas all patients with UCa history were excluded. N= number of analyzed patient probes.

## Slide 7
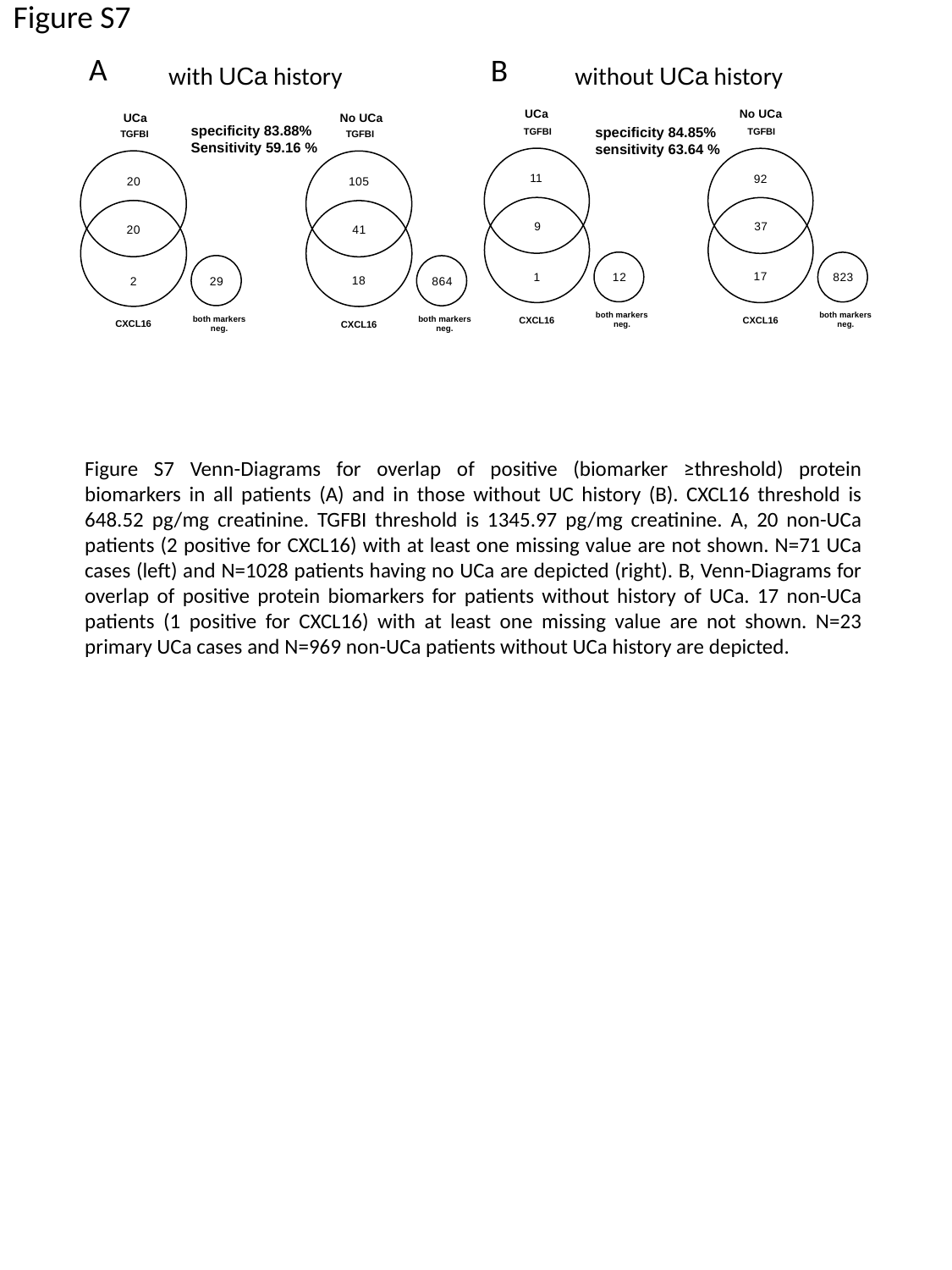

Figure S7
A
B
with UCa history
without UCa history
specificity 83.88%
Sensitivity 59.16 %
specificity 84.85%
sensitivity 63.64 %
Figure S7 Venn-Diagrams for overlap of positive (biomarker ≥threshold) protein biomarkers in all patients (A) and in those without UC history (B). CXCL16 threshold is 648.52 pg/mg creatinine. TGFBI threshold is 1345.97 pg/mg creatinine. A, 20 non-UCa patients (2 positive for CXCL16) with at least one missing value are not shown. N=71 UCa cases (left) and N=1028 patients having no UCa are depicted (right). B, Venn-Diagrams for overlap of positive protein biomarkers for patients without history of UCa. 17 non-UCa patients (1 positive for CXCL16) with at least one missing value are not shown. N=23 primary UCa cases and N=969 non-UCa patients without UCa history are depicted.

## Slide 8
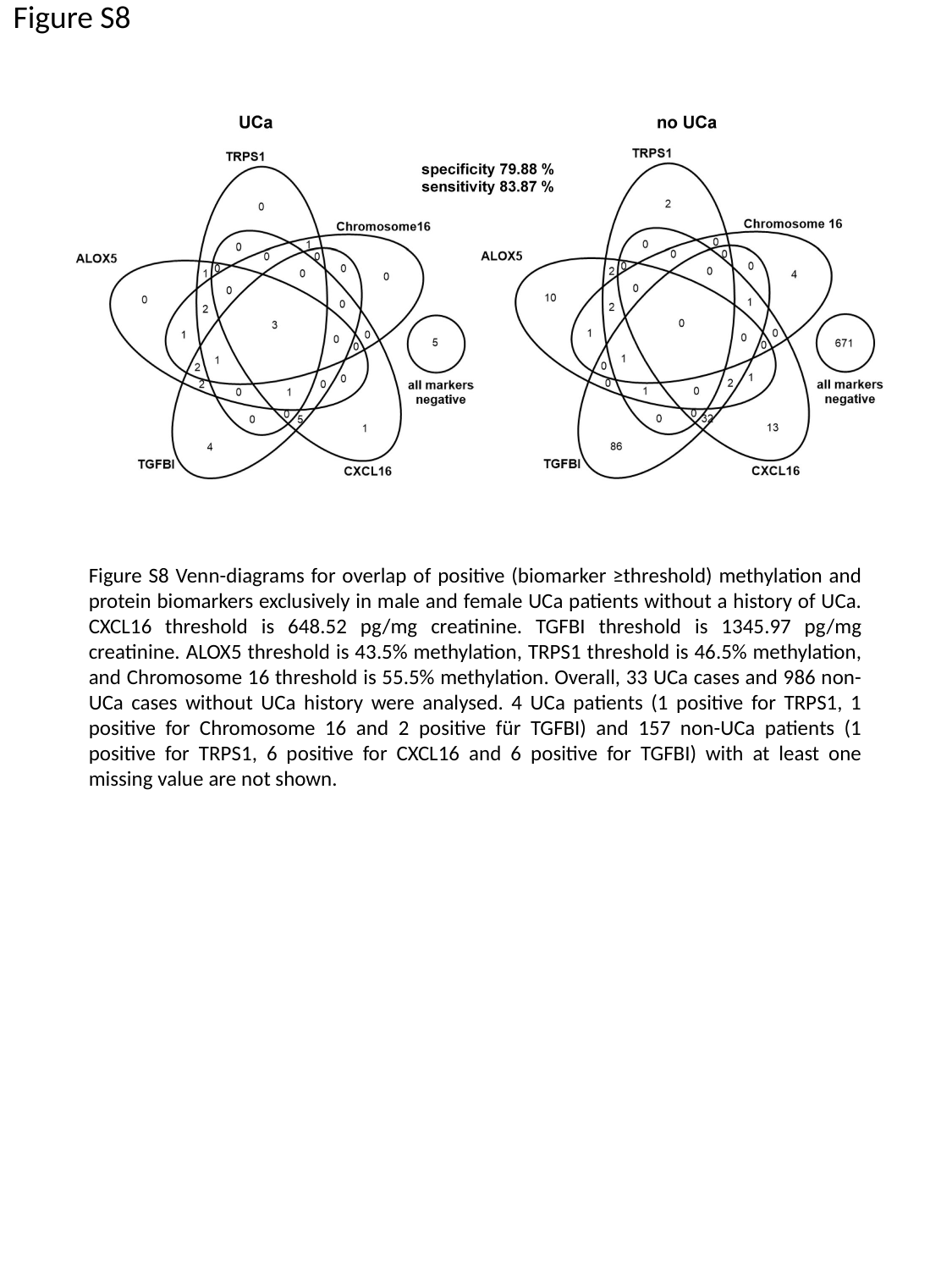

Figure S8
Figure S8 Venn-diagrams for overlap of positive (biomarker ≥threshold) methylation and protein biomarkers exclusively in male and female UCa patients without a history of UCa. CXCL16 threshold is 648.52 pg/mg creatinine. TGFBI threshold is 1345.97 pg/mg creatinine. ALOX5 threshold is 43.5% methylation, TRPS1 threshold is 46.5% methylation, and Chromosome 16 threshold is 55.5% methylation. Overall, 33 UCa cases and 986 non-UCa cases without UCa history were analysed. 4 UCa patients (1 positive for TRPS1, 1 positive for Chromosome 16 and 2 positive für TGFBI) and 157 non-UCa patients (1 positive for TRPS1, 6 positive for CXCL16 and 6 positive for TGFBI) with at least one missing value are not shown.

## Slide 9
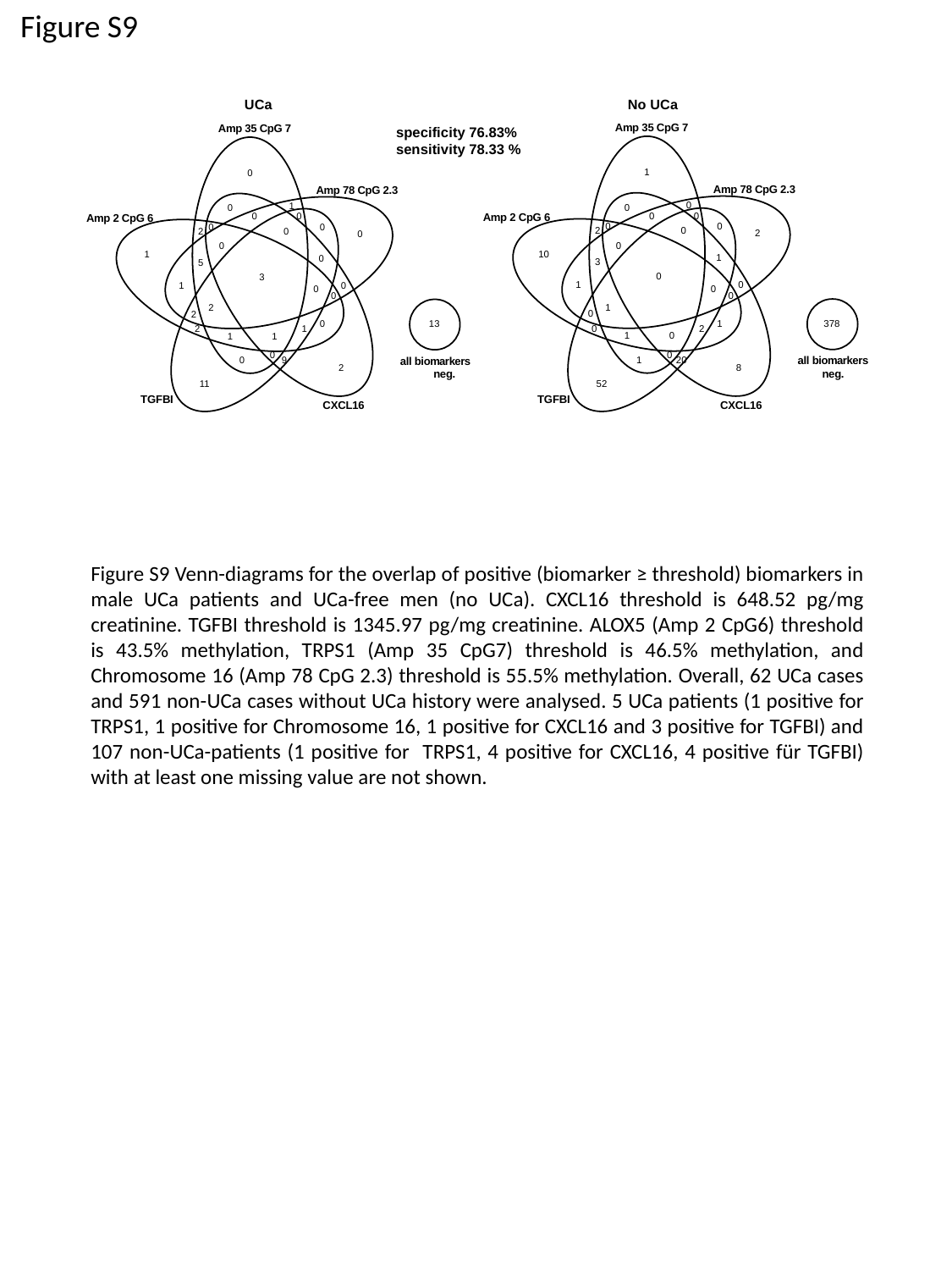

Figure S9
specificity 76.83%
sensitivity 78.33 %
Figure S9 Venn-diagrams for the overlap of positive (biomarker ≥ threshold) biomarkers in male UCa patients and UCa-free men (no UCa). CXCL16 threshold is 648.52 pg/mg creatinine. TGFBI threshold is 1345.97 pg/mg creatinine. ALOX5 (Amp 2 CpG6) threshold is 43.5% methylation, TRPS1 (Amp 35 CpG7) threshold is 46.5% methylation, and Chromosome 16 (Amp 78 CpG 2.3) threshold is 55.5% methylation. Overall, 62 UCa cases and 591 non-UCa cases without UCa history were analysed. 5 UCa patients (1 positive for TRPS1, 1 positive for Chromosome 16, 1 positive for CXCL16 and 3 positive for TGFBI) and 107 non-UCa-patients (1 positive for TRPS1, 4 positive for CXCL16, 4 positive für TGFBI) with at least one missing value are not shown.

## Slide 10
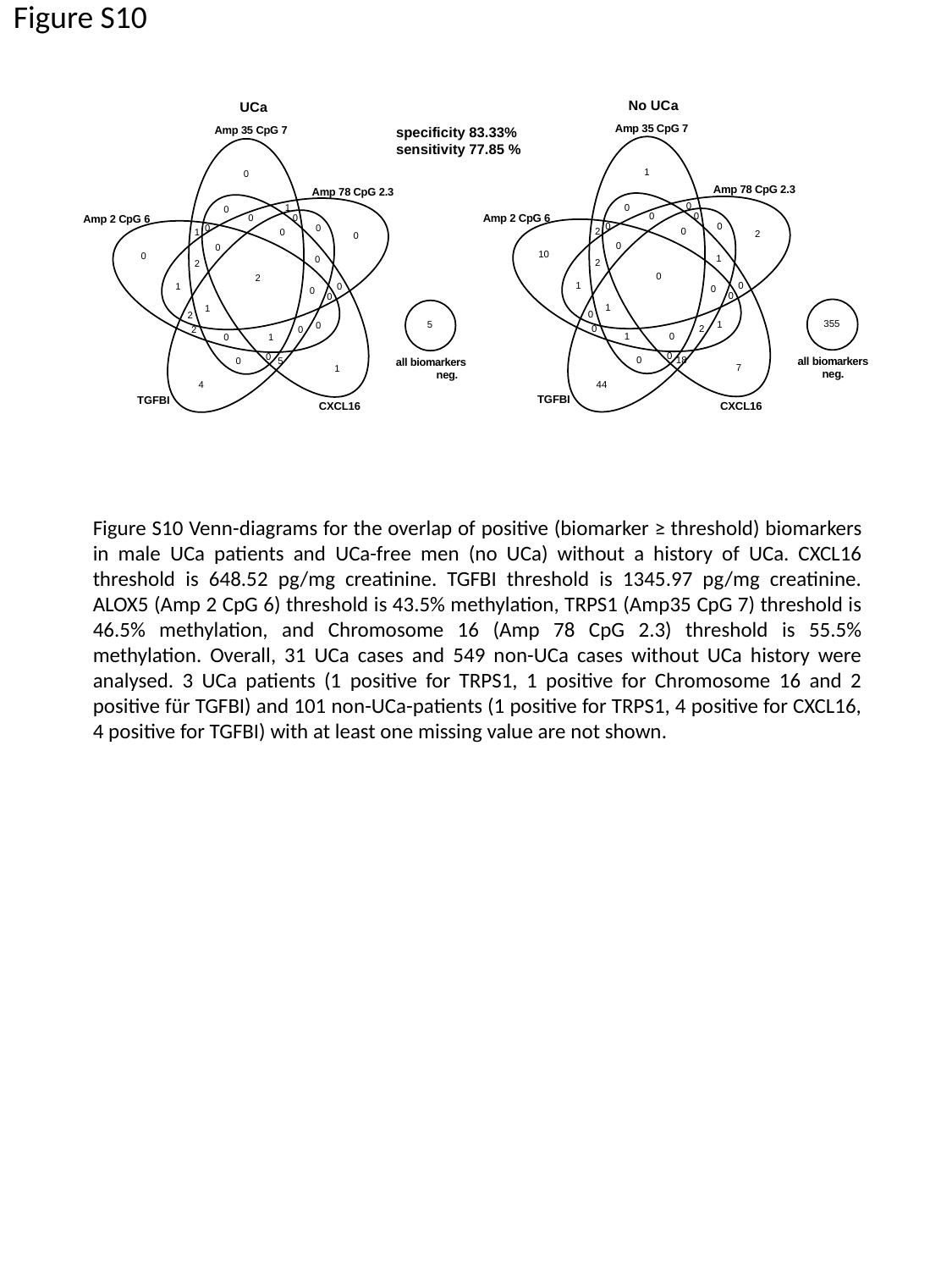

Figure S10
specificity 83.33%
sensitivity 77.85 %
Figure S10 Venn-diagrams for the overlap of positive (biomarker ≥ threshold) biomarkers in male UCa patients and UCa-free men (no UCa) without a history of UCa. CXCL16 threshold is 648.52 pg/mg creatinine. TGFBI threshold is 1345.97 pg/mg creatinine. ALOX5 (Amp 2 CpG 6) threshold is 43.5% methylation, TRPS1 (Amp35 CpG 7) threshold is 46.5% methylation, and Chromosome 16 (Amp 78 CpG 2.3) threshold is 55.5% methylation. Overall, 31 UCa cases and 549 non-UCa cases without UCa history were analysed. 3 UCa patients (1 positive for TRPS1, 1 positive for Chromosome 16 and 2 positive für TGFBI) and 101 non-UCa-patients (1 positive for TRPS1, 4 positive for CXCL16, 4 positive for TGFBI) with at least one missing value are not shown.

## Slide 11
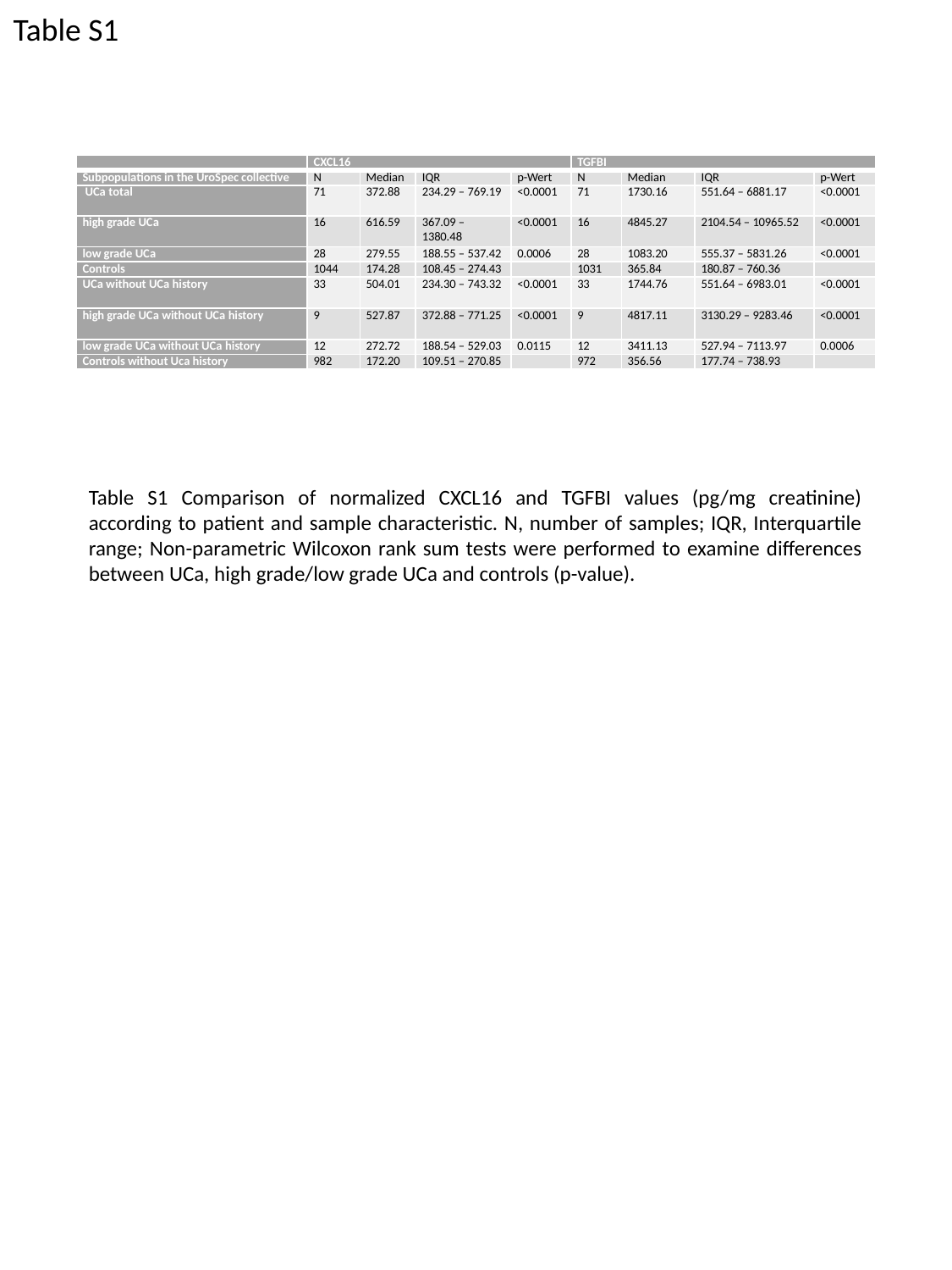

Table S1
| | CXCL16 | | | | TGFBI | | | |
| --- | --- | --- | --- | --- | --- | --- | --- | --- |
| Subpopulations in the UroSpec collective | N | Median | IQR | p-Wert | N | Median | IQR | p-Wert |
| UCa total | 71 | 372.88 | 234.29 – 769.19 | <0.0001 | 71 | 1730.16 | 551.64 – 6881.17 | <0.0001 |
| high grade UCa | 16 | 616.59 | 367.09 – 1380.48 | <0.0001 | 16 | 4845.27 | 2104.54 – 10965.52 | <0.0001 |
| low grade UCa | 28 | 279.55 | 188.55 – 537.42 | 0.0006 | 28 | 1083.20 | 555.37 – 5831.26 | <0.0001 |
| Controls | 1044 | 174.28 | 108.45 – 274.43 | | 1031 | 365.84 | 180.87 – 760.36 | |
| UCa without UCa history | 33 | 504.01 | 234.30 – 743.32 | <0.0001 | 33 | 1744.76 | 551.64 – 6983.01 | <0.0001 |
| high grade UCa without UCa history | 9 | 527.87 | 372.88 – 771.25 | <0.0001 | 9 | 4817.11 | 3130.29 – 9283.46 | <0.0001 |
| low grade UCa without UCa history | 12 | 272.72 | 188.54 – 529.03 | 0.0115 | 12 | 3411.13 | 527.94 – 7113.97 | 0.0006 |
| Controls without Uca history | 982 | 172.20 | 109.51 – 270.85 | | 972 | 356.56 | 177.74 – 738.93 | |
Table S1 Comparison of normalized CXCL16 and TGFBI values (pg/mg creatinine) according to patient and sample characteristic. N, number of samples; IQR, Interquartile range; Non-parametric Wilcoxon rank sum tests were performed to examine differences between UCa, high grade/low grade UCa and controls (p-value).

## Slide 12
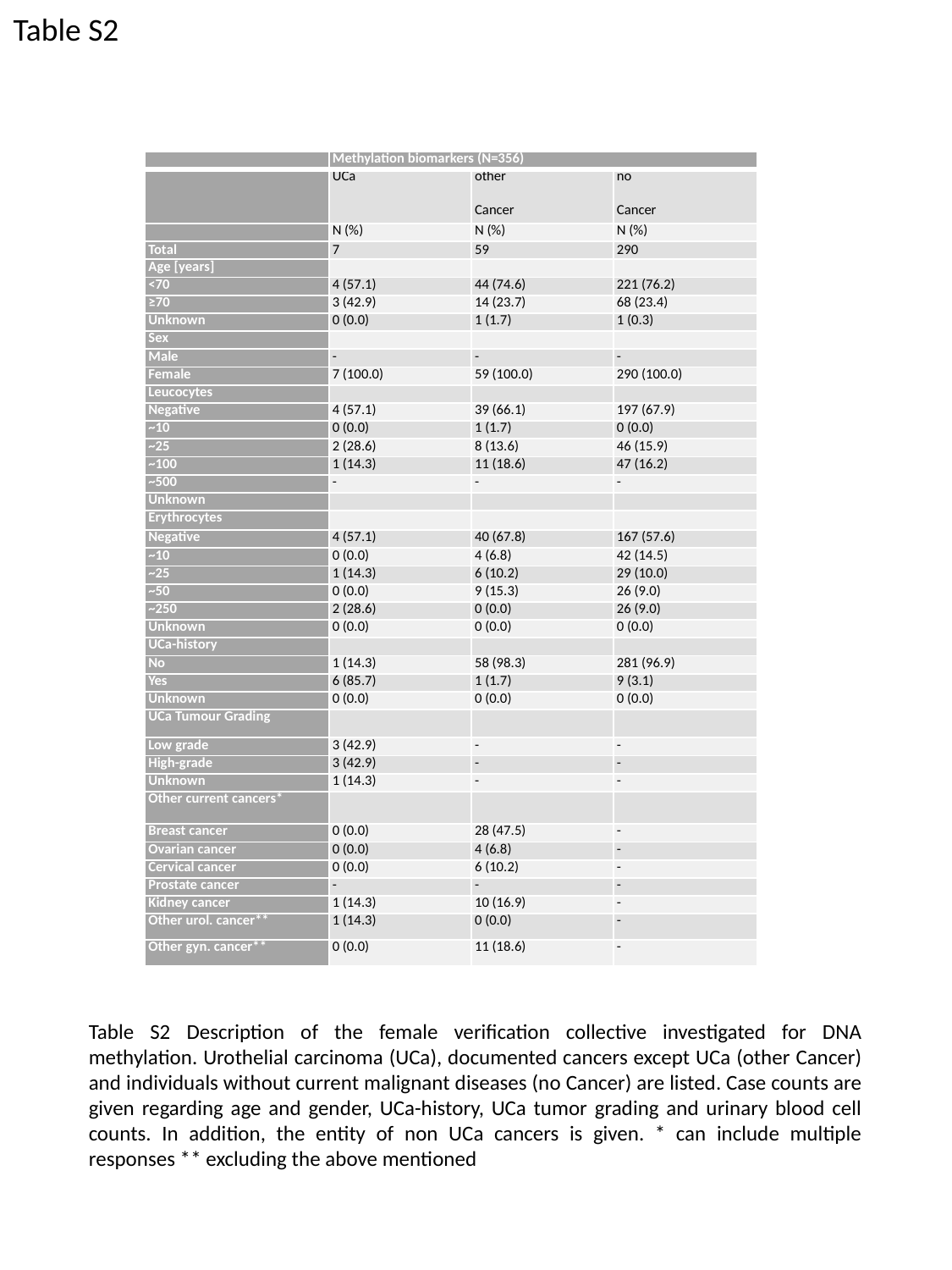

Table S2
| | Methylation biomarkers (N=356) | | |
| --- | --- | --- | --- |
| | UCa | other Cancer | no Cancer |
| | N (%) | N (%) | N (%) |
| Total | 7 | 59 | 290 |
| Age [years] | | | |
| <70 | 4 (57.1) | 44 (74.6) | 221 (76.2) |
| ≥70 | 3 (42.9) | 14 (23.7) | 68 (23.4) |
| Unknown | 0 (0.0) | 1 (1.7) | 1 (0.3) |
| Sex | | | |
| Male | - | - | - |
| Female | 7 (100.0) | 59 (100.0) | 290 (100.0) |
| Leucocytes | | | |
| Negative | 4 (57.1) | 39 (66.1) | 197 (67.9) |
| ~10 | 0 (0.0) | 1 (1.7) | 0 (0.0) |
| ~25 | 2 (28.6) | 8 (13.6) | 46 (15.9) |
| ~100 | 1 (14.3) | 11 (18.6) | 47 (16.2) |
| ~500 | - | - | - |
| Unknown | | | |
| Erythrocytes | | | |
| Negative | 4 (57.1) | 40 (67.8) | 167 (57.6) |
| ~10 | 0 (0.0) | 4 (6.8) | 42 (14.5) |
| ~25 | 1 (14.3) | 6 (10.2) | 29 (10.0) |
| ~50 | 0 (0.0) | 9 (15.3) | 26 (9.0) |
| ~250 | 2 (28.6) | 0 (0.0) | 26 (9.0) |
| Unknown | 0 (0.0) | 0 (0.0) | 0 (0.0) |
| UCa-history | | | |
| No | 1 (14.3) | 58 (98.3) | 281 (96.9) |
| Yes | 6 (85.7) | 1 (1.7) | 9 (3.1) |
| Unknown | 0 (0.0) | 0 (0.0) | 0 (0.0) |
| UCa Tumour Grading | | | |
| Low grade | 3 (42.9) | - | - |
| High-grade | 3 (42.9) | - | - |
| Unknown | 1 (14.3) | - | - |
| Other current cancers\* | | | |
| Breast cancer | 0 (0.0) | 28 (47.5) | - |
| Ovarian cancer | 0 (0.0) | 4 (6.8) | - |
| Cervical cancer | 0 (0.0) | 6 (10.2) | - |
| Prostate cancer | - | - | - |
| Kidney cancer | 1 (14.3) | 10 (16.9) | - |
| Other urol. cancer\*\* | 1 (14.3) | 0 (0.0) | - |
| Other gyn. cancer\*\* | 0 (0.0) | 11 (18.6) | - |
Table S2 Description of the female verification collective investigated for DNA methylation. Urothelial carcinoma (UCa), documented cancers except UCa (other Cancer) and individuals without current malignant diseases (no Cancer) are listed. Case counts are given regarding age and gender, UCa-history, UCa tumor grading and urinary blood cell counts. In addition, the entity of non UCa cancers is given. * can include multiple responses ** excluding the above mentioned

## Slide 13
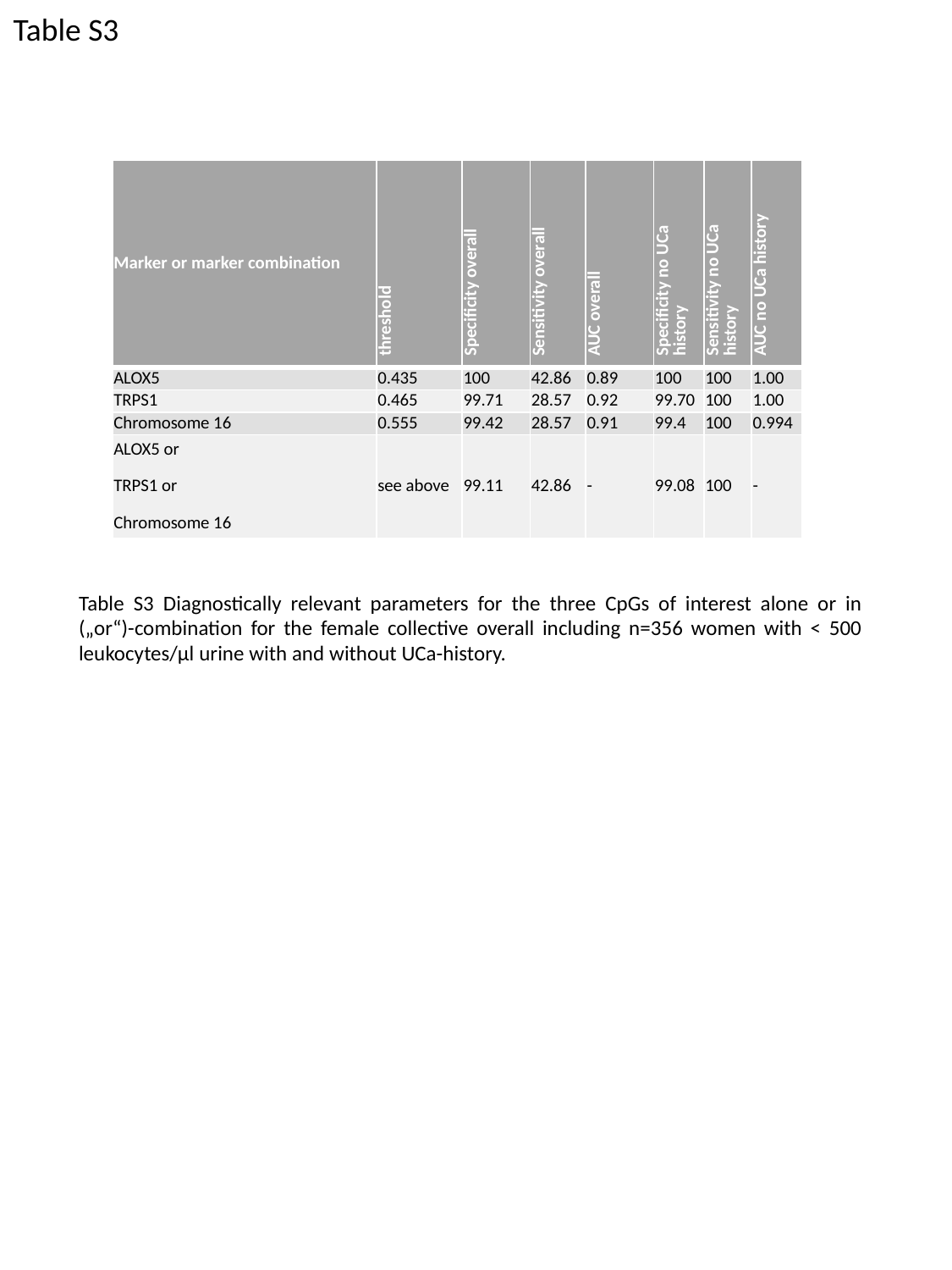

Table S3
| Marker or marker combination | threshold | Specificity overall | Sensitivity overall | AUC overall | Specificity no UCa history | Sensitivity no UCa history | AUC no UCa history |
| --- | --- | --- | --- | --- | --- | --- | --- |
| ALOX5 | 0.435 | 100 | 42.86 | 0.89 | 100 | 100 | 1.00 |
| TRPS1 | 0.465 | 99.71 | 28.57 | 0.92 | 99.70 | 100 | 1.00 |
| Chromosome 16 | 0.555 | 99.42 | 28.57 | 0.91 | 99.4 | 100 | 0.994 |
| ALOX5 or TRPS1 or Chromosome 16 | see above | 99.11 | 42.86 | - | 99.08 | 100 | - |
Table S3 Diagnostically relevant parameters for the three CpGs of interest alone or in („or“)-combination for the female collective overall including n=356 women with < 500 leukocytes/µl urine with and without UCa-history.
